# Structural Effects of Aluminum and Iron Occupancy in Minerals of the Jarosite-Alunite Solid Solution

**DOI:** 10.1021/acsearthspacechem.3c00174

**Published:** 2024-01-25

**Authors:** Andrew R. C. Grigg, Luiza Notini, Ralf Kaegi, Laurel K. ThomasArrigo, Ruben Kretzschmar

**Affiliations:** † Soil Chemistry Group, Institute of Biogeochemistry and Pollutant Dynamics, Department of Environmental Systems Science, ETH Zurich, Universitätstrasse 16, CHN, CH-8092 Zurich, Switzerland; ‡ Eawag, Swiss Federal Institute of Aquatic Science and Technology, Überlandstrasse 133, Dübendorf, CH-8600 Dübendorf, Switzerland; § Environmental Chemistry Group, Institute of Chemistry, University of Neuchâtel, Avenue de Bellevaux 51, CH-2000 Neuchâtel, Switzerland

**Keywords:** Raman spectroscopy, Mössbauer spectroscopy, energy dispersive X-ray spectroscopy, X-ray diffraction, atom substitution

## Abstract

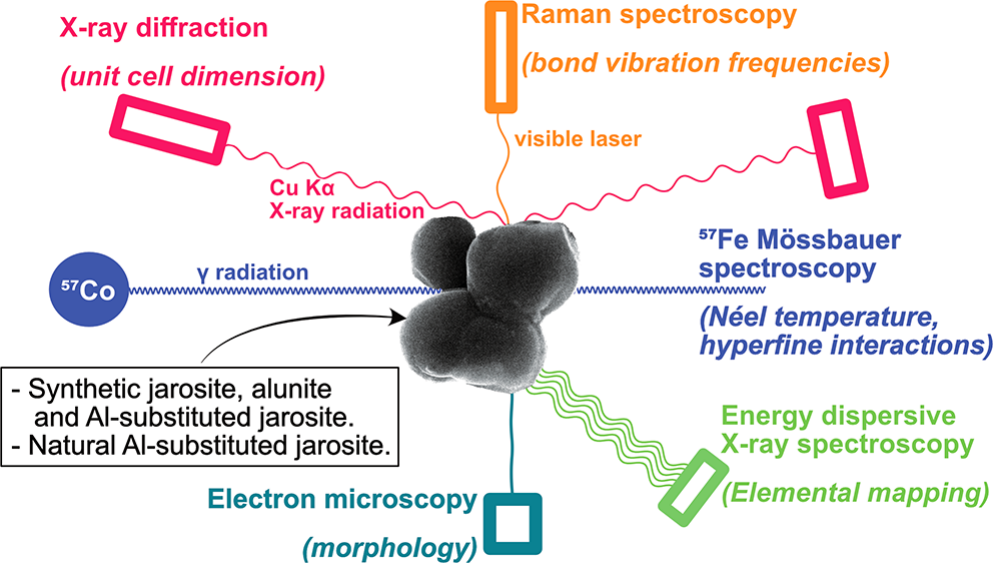

The alunite supergroup of minerals contains several hydroxysulfate mineral phases that commonly occur in acidic natural and engineered environments. The main division of the mineral supergroup defines two minerals, jarosite and alunite, based on the relative structural occupancy by Al or Fe, respectively. However, intermediate members of the jarosite-alunite solid solution have not been extensively characterized, especially in the environment. Here, we link the mineral unit cell sizes measured by X-ray diffraction, peak shifts in Raman spectra, fitting parameters in Mössbauer spectroscopy, and elemental quantification by EDX spectroscopy to known amounts of Al substitution in two synthetic series of Al-substituted jarosite (up to Al-for-Fe substitution of 9.5%) and unknown Al substitution in a natural jarosite isolated from an acid sulfate soil. Strong correlations were observed between the Al substitution of the jarosite samples and unit cell size, position of several vibrational peaks in Raman spectroscopy, and the temperature of magnetic ordering. In addition, elemental mapping provided a robust way to characterize the Al content of jarosite. As the techniques were effective in quantifying the Al or Fe content of jarosite-alunite supergroup mineral samples, without the need for sample dissolution, the findings support the application of these spectroscopy techniques to characterize natural jarosite-alunite samples. Using these techniques, we demonstrate at least 5% Al-for-Fe substitution in a jarosite sample from an acid sulfate soil. Application to environmental samples is especially useful in cases where it is otherwise difficult to directly measure the Al content of a mineral sample or when Al-for-Fe substitution influences the spectral responses to substitution at other sites in the crystal structure.

## 1 Introduction

Jarosite and alunite are common hydroxysulfate minerals found in various natural and engineered systems. Both minerals are typical of acid sulfate environments such as acid-rock drainage and supergene weathered pyrite deposits.^1,2^ Jarosite is often diagnostic of acid sulfate soils (ASS).^3,4^ Alunite may also form in ASS, although it is more commonly a product of hypogene processes.^2,5^ Jarosite and alunite have been found in diverse niche environments such as hypersaline lakes,^6^ Antarctic ice,^7^ and the surface of Mars.^8,9^ Furthermore, jarosite is used extensively in hydrometallurgical industries.^10,11^ As isostructural members of the alunite supergroup of minerals, jarosite- and alunite-subgroup minerals both have the general formula AB_3_(TO)_4_(OH)_6_. The primary division of the mineral supergroup is based on the predominance of Fe or Al at the B-site (jarosite or alunite, respectively),^11,12^ and a complete solid solution series (SSS) between jarosite and alunite occurs according to Vegard’s law.^13^ Minor B-site substitution by Sb^5+^, Ga^3+^, In^3+^, Tl^3+^, and various divalent metal cations have been reported.^11,14^ The A-site is most commonly occupied by K^+^, Na^+^, H_3_O, or NH_4_^+^, but may also contain other large cations such as Rb^+^, Ag^+^, Tl^+^, Ca^2+^, Sr^2+^, Ba^2+^, Pb^2+^, Hg^2+^, Bi^2+^, or rare earth elements.^2,11,15^ The T-site is most commonly occupied by S^6+^, but may also contain P^5+^ or As^5+^.^2,15,16^ Here, we refer to KFe_3_(SO)_4_(OH)_6_ and KAl_3_(SO)_4_(OH)_6_ as jarosite and alunite, respectively, while KFe_*x*_Al_y-x-z_(SO_4_)_2_(OH)_6_ is called Al-substituted jarosite for *x* > 1.5, *y* < 3 and z (site deficiency or substitution by other cations) < 1.5.

Intermediate members of the jarosite-alunite SSS have been identified in various environments, such as a hypersaline Australian lake (20% Al-for-Fe substituted jarosite and 8% Fe-for-Al substituted alunite),^6^ an acidic Spanish lake (various degrees of substitution along SSS),^17^ late Miocene to late Pliocene acid sulfate sediments (2:1 Al:Fe),^18^ on White Island Volcano, New Zealand (16% Al-for-Fe substitution),^19^ various hypergene deposits (up to 5.1% Al-for-Fe substitution from Lone Tree Mine, Nevada),^20^ and in hydrometallurgical wastes (up to 1:24 Al:Fe).^21^ However, the measurement of Al substitution based on elemental analysis of dissolved minerals is challenging when the sample material contains other mineral phases. For example, Al substitution in jarosite found in clayey soils can be difficult to measure because analytical methods may not adequately distinguish Al substitution in jarosite from Al in coexisting clays or soluble Al mineral phases.^22,23^ Such analytical challenges may have contributed to a long-standing view that intermediate members of the jarosite-alunite SSS are not common in the environment.^24,25^ In fact, Al substitution of jarosite can be extensive and may control the availability of Al in some acid sulfate environments.^17,23^

Among the studies of synthetic alunite supergroup minerals, only a small number have characterized intermediate members of the jarosite-alunite SSS. Synthesis studies show that Fe is incorporated at the B-site of jarosite-alunite minerals preferentially to Al, but higher pH, temperature, and pressure during synthesis increase Al occupancy at the B-site.^24,26^ The primacy of Fe at low pH is caused by the lower enthalpies of the formation of Fe-rich jarosite-alunite minerals compared with Al-rich members of the SSS^27^ and contrasting hydrolysis constants of Fe^3+^ and Al^3+^.^24^ X-ray diffraction has been extensively used to characterize minerals from synthetic jarosite-alunite SSS, showing that increased Al-for-Fe substitution corresponds to a decrease in the *a* parameter of the unit cell.^24,26,27^ Various spectroscopy techniques have also been applied. At the micrometer scale, energy-dispersive X-ray spectroscopy (EDX) can be used to quantify the Al and Fe content of crystals of particular mineral phases in isolation.^17,18,28^ On bulk samples, Raman spectroscopy is particularly suitable for the characterization of many sulfate-containing minerals, such as alunite supergroup minerals.^29^ Substitution at the A-site has a consistent effect on the position of Raman spectral peaks associated with some FeO and SO_4_ vibrational bands,^29−31^ and the radius of the ion at the B-site has an impact on all peak positions.^32^ The effect of partial B-site substitution in jarosite-alunite minerals is less well documented. Another spectroscopic method with application to Fe minerals such as jarosite is ^57^Fe Mössbauer spectroscopy,^28,33−35^ although the effect of substitution on the Mössbauer parameters of jarosite-alunite minerals is currently poorly documented. Additional measurements of jarosite-alunite group minerals by Raman and Mössbauer spectroscopy are required to link elemental substitutions to changes of spectral parameters.^35,36^

In this study, our aim was to investigate the effect of Fe and Al occupancy on the unit cell and spectroscopic parameters of jarosite. In contrast to previous studies,^13,27^ the motivation for our study was to explore whether spectroscopic and X-ray diffraction (XRD) techniques can be used to characterize the elemental composition of jarosite-alunite minerals in the laboratory and in nature without requiring dissolution of the mineral. For this purpose, two jarosite-alunite series were synthesized using different methods: A hydrothermal method to ensure a rapid high-yield synthesis with high Al uptake and a room temperature series to investigate the behavior of crystals formed at temperatures similar to those in natural soil. Both series were analyzed by Mössbauer spectroscopy, Raman spectroscopy, analytical electron microscopy, and XRD. We, then, estimated the composition of a natural jarosite from an ASS in Thailand using these techniques. This study advances the characterization of the Fe-rich members of the jarosite-alunite series, particularly focusing on samples containing Al-for-Fe substitution up to 9.5% to match levels of Fe-for-Al substitution that are relevant for jarosite found in ASS. This study defines the effects of Al substitution on Raman and Mössbauer spectra; refines the application of XRD to measure the relationship between the unit cell size and Al-for-Fe substitution; and provides a critical assessment of the practicality of the techniques to assess the Al-for-Fe substitution in alunite-jarosite minerals.

## 2 Methods

### 2.1 Synthesis of Jarosite-Alunite Minerals

One series of jarosite-alunite minerals (henceforth “HT-Jrs”) was synthesized from a hydrothermal method adapted by Driscoll and Leinz.^37^ The hydrothermal method was chosen as it maximizes the jarosite yield and Al incorporation of the synthesis. To begin, a solution of Fe(III) was made by dissolving Fe(0) (10 μm metal powder, EMSURE analysis grade, Merck) in 2 M H_2_SO_4_ (95–97% reagent grade, Sigma-Aldrich) with agitation using a submerged magnetic stirring bar, oxidizing with excess H_2_O_2_ (35%, Merck) to a final Fe concentration of 0.9 M and filtering out particulate Fe with a 0.22 μm nylon syringe filter (BGB, Switzerland). Then, a series of solutions were produced with Al/(Fe+Al) between 0 and 0.4 and constant (Fe + Al)/SO_4_^2–^ by mixing selected quantities of the Fe(III) solution and a saturated solution of AlK(SO_4_)_2_·12H_2_O (Normapur reagent grade, VWR). Additionally, alunite (“HT-Alu”) was synthesized by increasing Al/(Fe+Al) to 1 (i.e., replacing all Fe(III) with Al(III) from AlK(SO_4_)_2_·12H_2_O solution). The pH of the solutions was raised to 2.50 ± 0.05 with 2 M KOH (Pellets extra pure, Merck), which simultaneously increased the K^+^ concentration of all solutions to a stoichiometric excess for jarosite. The final volume adjustment to 25 mL was carried out with ultrapure water (UPW; Millipore, > 18.2 MΩ cm). The solutions were heated in Teflon vessels inside a steel heating block at 140 °C for 5 h. After cooling, the supernatant was decanted and discarded. Solids from the Teflon vessels were rinsed by repeated resuspension in UPW, centrifugation (3575 *g*), and decanting, until the conductivity of the supernatant was <100 μS/cm. The minerals were dried for 24 h at 60 °C, gently homogenized with mortar and pestle, and stored in amber glass vials in a desiccator.

A second jarosite-alunite SSS (henceforth “RT-Jrs”) was synthesized from solutions prepared in the same way as for the hydrothermal jarosite but reacted in 50 mL centrifuge tubes on an overhead shaker at room temperature for 16 days. Precipitates were rinsed, dried, prepared, and stored as mentioned above. This method was chosen, as the lower temperature and longer crystallization time are more relevant to acid sulfate soil environments at the earth’s surface. Increasing Al concentrations were associated with decreasing yield, and alunite could not be synthesized by this method.

A sample of natural jarosite was collected from an ASS in Chachoengsao province, Thailand. The soil (photograph in Figure S1) was a Hydric Vertic Anthrosol (World Reference Base for Soils^4^) from the Rangsit Soil Series (Thai soil taxonomy^38^) and is in regular use as a rice paddy. Soils of the Rangsit Series initially formed from a mixture of marine and riverine sediments under brackish water but have been cultivated for some time. Seasonal flux of the water table has led to the formation of jarosite mottles in the profile.^38^ Soil samples from between 68 and 135 cm depth (the zone in which jarosite mottles occurred in the soil profile) were dried in an oven at 30 °C. Jarosite was separated from other mineral phases by scraping light yellow minerals from mottles with a scalpel. The isolated natural jarosite was stored in an amber glass vial in a desiccator.

### 2.2 Characterization

#### 2.2.1 Electron Microscopy

All electron microscopy (EM) was carried out on approximately 2 mg of the mineral sample that was resuspended in UPW and drop-deposited onto 200-mesh Cu grids with a holey carbon support film (SPI supplies, USA). Secondary electron (SE) images were obtained on a scanning transmission electron microscope (2700Cs, Hitachi). Energy-dispersive X-ray spectroscopy (EDX) and high-angle annular dark field (HAADF) images were produced on an FEI Talos F200X S/TEM microscope.

#### 2.2.2 Elemental Composition

Synthetic and natural mineral subsamples were dissolved in 4 M HCl.^39^ The concentrations of Fe, S, and Al were measured by inductively coupled plasma optical emission spectrometry (ICP-OES; 5100, Agilent Technologies, USA). Concentrations of K were measured by atomic absorption spectrometry (AAS; 240FS spectrometer, Agilent Technologies, USA) at 766.5 nm with sample atomization in an air-acetylene flame. Organic carbon was measured by combustion of an undissolved mineral sample (Vario EL CHNS analyzer, Elementar, Germany).

#### 2.2.3 X-ray Diffraction Analysis

Powder XRD measurements were performed on samples that were resuspended in ethanol, transferred onto polished Si wafers (711 cut, Sil’tronix Silicon Technologies, France), and allowed to dry in place. Samples were measured using a Bruker D8 Advance (Bruker, USA) in Bragg–Brentano geometry using Cu Kα radiation (λ_Kα1_ = 1.540562 Å, λ_Kα2_ = 1.544398 Å, 40 kV, 40 mA) and a high-resolution energy-dispersive 1D detector (LYNXEYE). Measurements were taken between 10° and 70° 2θ with a step size of 0.02° 2θ and measurement time of 10 s/step.

Rietveld quantitative phase analysis was performed using TOPAS software (Version 5, Bruker, USA). The jarosite was fit with previously published Crystallographic Information Files (CIF).^40^ Partial replacement of O for K and Al for Fe in the model, to take account of potential hydronium and Al substitution, did not improve the fitting and was not included in the final model. The models were improved by fitting the preferred orientation on the jarosite (0 0 3) direction and vertical sample displacement. The crystallite size was estimated from diffraction line widths using *TOPAS* to calculate the LVol-IB by combining Lorentzian and Gaussian profiles. Instrumental peak broadening was taken into account using a LaB_6_ standard reference material (NIST SRM 660c) with a mean volume-weighted domain size of 0.8 μm.

#### 2.2.4 Raman Spectroscopy

Following XRD analysis, the minerals on silicon wafers were measured by micro-Raman spectroscopy with an inVia 2 confocal Raman Microscope (Renishaw, UK). All samples were measured with a 532 nm laser through a 50x microscope objective using laser power below 1% to avoid beam damage in the samples. On each sample, a low-density grating (1800 mm/l) was used for an overview spanning as many wavelengths as possible (maximum domain size), and a higher density grating (3000 mm/l) was used to produce high-precision spectra for the determination of vibration band positions. Calibration to the 520.5 cm^–1^ line of silica was completed before each sample measurement and measurement time was limited to <18 h to avoid calibration drift. Between 1500 and 2500 independent 8-s-long measurements were averaged to produce a spectrum of synthetic mineral samples, and 900–1000 independent measurements were taken for the natural jarosite samples, with 15 accumulations of 4 seconds each at every location. The accumulation method was used to avoid the saturation of the detector by fluorescence. Spectra were processed to remove spikes attributed to cosmic rays, subtract the baseline, and average the results from each sample (Wire 5.2 software, Renishaw plc, UK). Peak locations were measured using the “curve fit” function in Wire 5.2 software (Renishaw plc, UK) to take account of the total peak shape and to avoid biases introduced by noise or limited resolution of the measured spectra.

#### 2.2.5 Mössbauer Spectroscopy

Samples for Mössbauer analysis were deposited on a 0.22 μm MCE filter by passing a UPW suspension of the sample mineral through the filter. The mineral sample was air-dried on the filter and sealed in polyimide film tape (3M Company, USA). Spectra were measured on a WSS-10 spectrometer (WissEL GmbH, Germany) in transmission using a ^57^Co/Rh γ-radiation source. A liquid-He cryostat (Janis Research, USA) was used to cool samples to a series of temperatures between 77 and 4.2 K, each of which was held constant throughout the measurement period. Spectra were fit using extended Voigt-based fitting (xVBF) with Recoil software.^41,42^ The velocity scale was calibrated to an α-Fe foil. The Lorentzian full width at half-maximum of all fits was fixed to 0.135 mm/s, as measured on the Fe foil calibration.

## 3 Results and Discussion

### 3.1 Particle Properties

**Figure 1:**
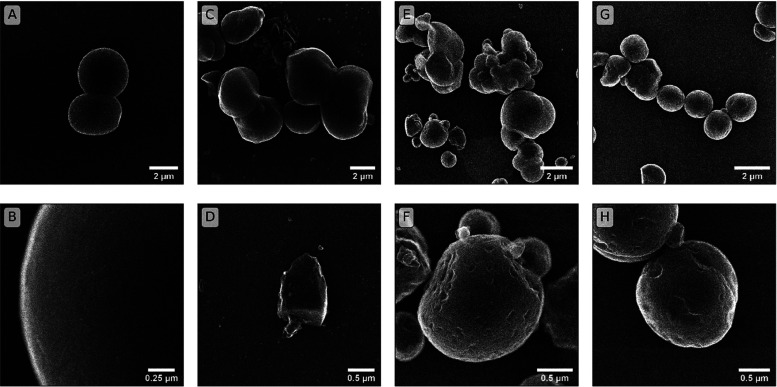
Comparison of secondary electron (SE) images from HT and RT jarosite samples. Panels A and B are SE images of HT-Jrs without Al substitution. Panels C and D are images of HT jarosite with 7.3% Al-for-Fe substitution. Panels E and F are of RT-Jrs without Al substitution. Panels G and H are images of RT-Jrs with 0.6% Al-for-Fe substitution. Elemental distribution maps of Fe, S, K, and Al derived from EDX analyses of samples of 7.3% Al-for-Fe substitution HT jarosite, unsubstituted RT-Jrs and 0.6% Al-for-Fe substituted RT-Jrs, are presented in Figures S2–S4, respectively. Additionally, SE imagery and elemental distribution maps of Fe, S, K, Al, and Si for natural jarosite are available in Figures S5 and S6.

The synthetic jarosite-alunite minerals had a globular to spherical particle morphology. Unsubstituted HT-Jrs occurred as spheres with diameters of approximately 2–5 μm (Figure 1A,B), while 7.3% Al-for-Fe substituted HT-Jrs displayed less regular spherical shapes, intermixed with crystals of angular form (Figure 1C,D). The intergrowth of spherical particles in RT-Jrs also produced a globular form. Particles of approximately 0.5–2 μm diameter occurred in RT-Jrs samples, with prominent surface pitting in both unsubstituted RT-Jrs (Figure 1E,F) and 0.6% Al-for-Fe substituted RT-Jrs (Figure 1G,H). The particles seen in SE images were made up of many crystallites, with sizes within the range of 84–94 nm for HT-Jrs and 75–82 nm for RT-Jrs (LVol-IB estimated by XRD; Table S4). Globular to spherical forms have been previously observed in synthetic jarosite,^13,43^ although decreasing Al content was associated with less regular spherical shapes and more particle intergrowth.^13^ Differences between the two synthesis methods, such as temperature, pressure, and length of time, likely explained the different size and morphology of the product particles. However, the crystallite sizes in HT-Jrs and RT-Jrs were similar and indicated that crystalline mineral products formed in both synthesis series.

**Figure 2:**
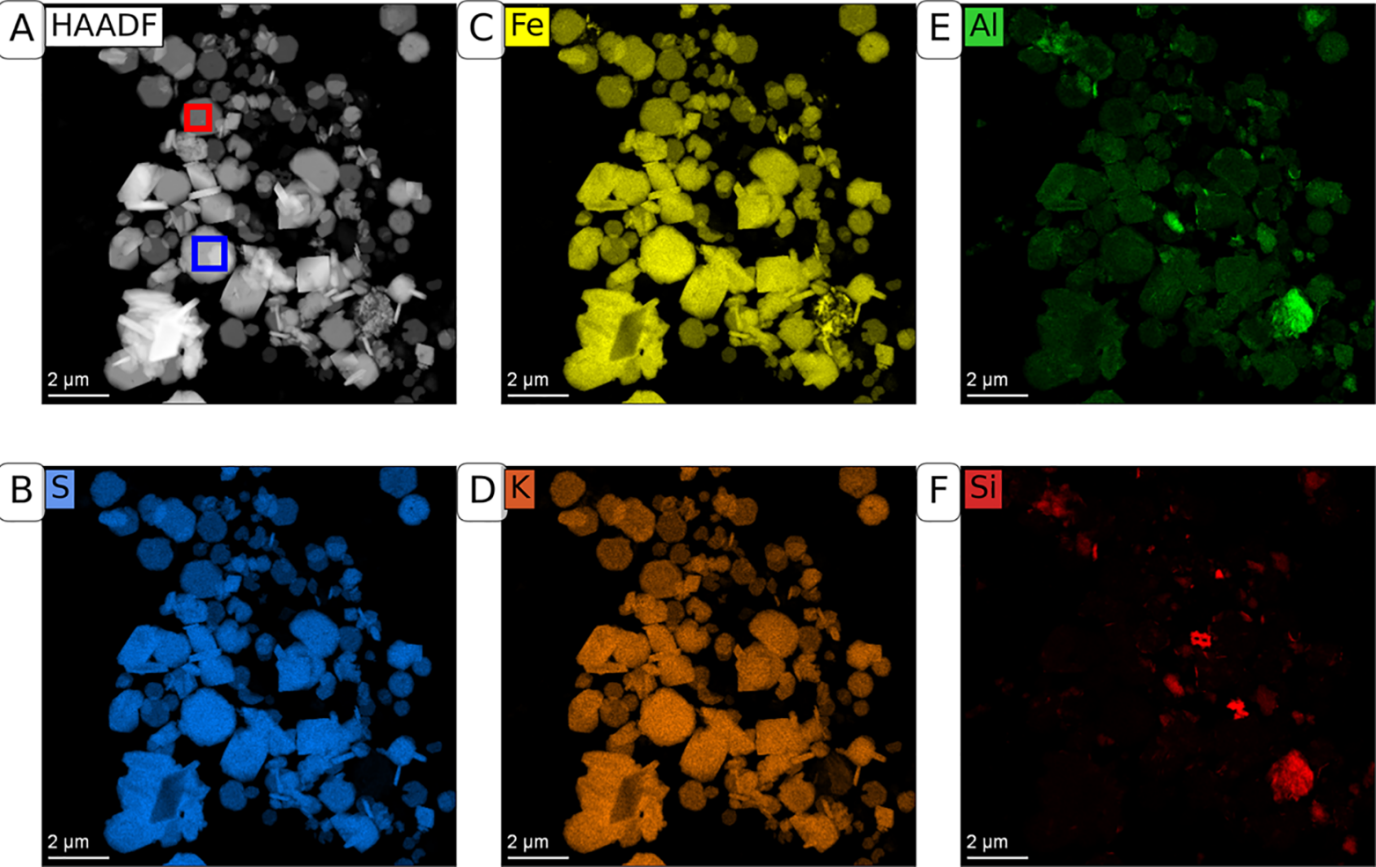
Elemental distribution maps of a sample of natural jarosite from an acid sulfate soil in Thailand, measured by EDX. (A–F) High angular dark-field image (HAADF), distribution of S, distribution of Fe, distribution of K, distribution of Al, and distribution of Si, respectively. The EDX spectra for quantification of element ratios (plotted in Figure S5, quantification summarized in Table S1) were measured in the red and blue boxes indicated in Panel A.

In contrast to the two synthetic jarosite series, the natural jarosite samples (Figures 2, S5 and S6) contained different morphological types, ranging from polyhedral to tabular. The polyhedral habit may reflect a cubic or pseudocubic crystal with partially or fully developed octahedral faces, as has been observed in previous studies of natural jarosite.^23,44^ Individual clay particles present in the natural samples can only partly explain the diversity of particle forms seen in the SE images of natural jarosite. This is supported by elemental distribution maps derived from EDX analysis (Figure 2) showing that key elements of jarosite (K, Fe, and S) are evenly distributed in particles of different morphologies and that these particles contain only negligible amounts of Si. The diameter of the natural jarosite particles was approximately 1–2 μm, which was slightly smaller than the synthetic jarosite samples. Nonetheless, the crystallite size of 83 nm (LVol-IB estimated by XRD) was similar to the crystallite size of the synthetic jarosite samples (Table S4).

The color of HT-Jrs minerals was “yellow”, ranging from 2.5Y 7/8 for unsubstituted to 2.5Y 8/8 for 9.5%-Al-substituted (Munsell colors, Table S2). Alunite was “white”. Minerals in the RT-Jrs series were also “yellow”, ranging from 5Y 8/6 for unsubstituted jarosite (chroma was higher than any available physical reference) to 2.5Y 8/6 for 3.0% Al substitution and to 2.5 Y 7/8 for 8.0% Al-substituted RT-Jrs (Table S2). The natural jarosite sample was “pale yellow” (7.5Y 8/3).

### 3.2 Elemental Composition

**Figure 3:**
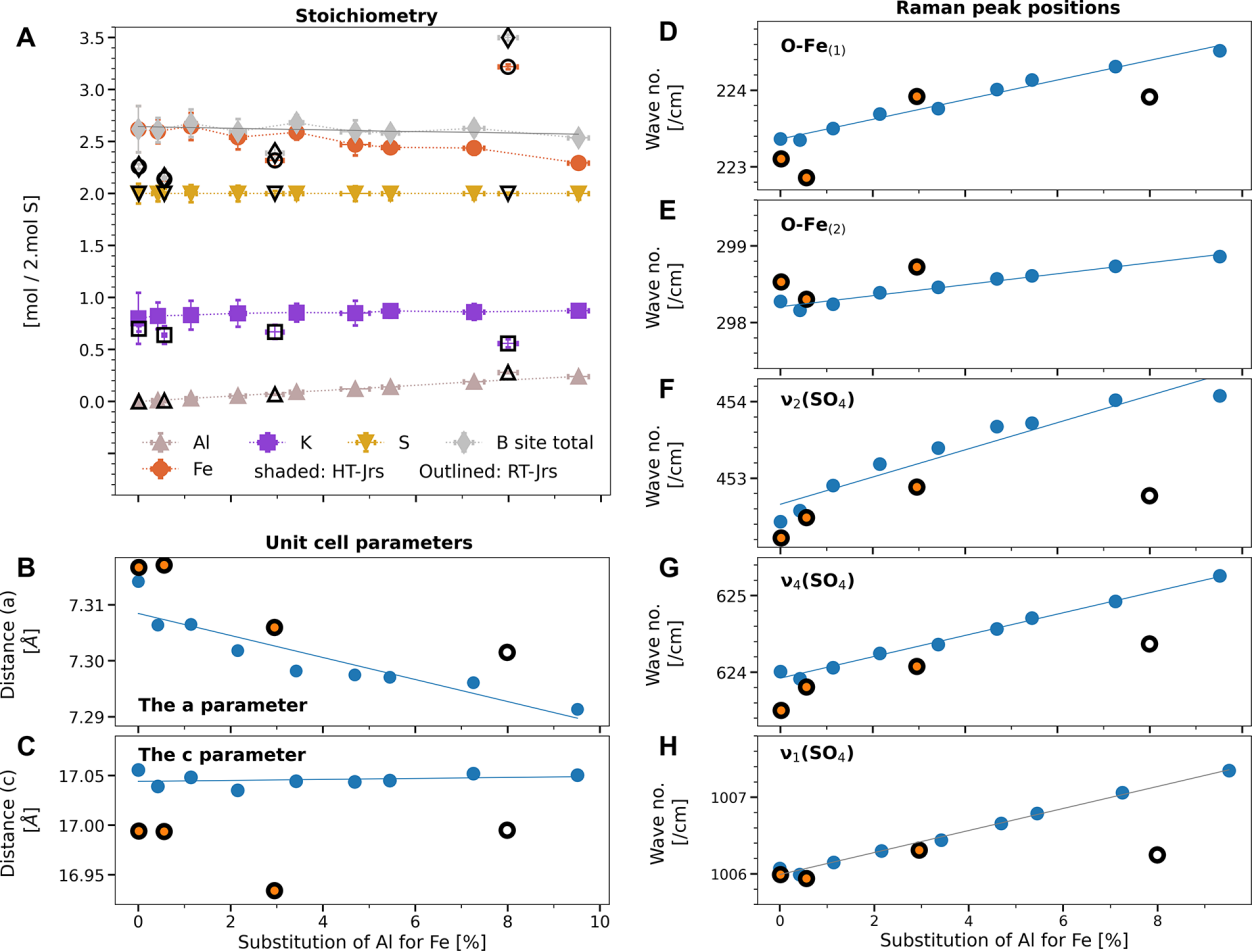
Properties of the HT and RT Al-jarosite synthesis series (alunite omitted). (A) Stoichiometry of all elements, normalized to S. HT-Jrs results are plotted with single color points, whereas RT-Jrs results are plotted with black outlines. Measured values are found in Table S1. (B,C) Plotted unit cell dimensions based on Rietveld fitting of XRD patterns. All XRD fits are presented in Figures S7–S9. The HT-Jrs series are plotted in blue and the RT-Jrs series with a black outline. The RT sample without fill color does not fit the regression due to the formation of impurities during synthesis. (D–G) Selected peak positions from Raman spectra with fitted regression through HT points (blue points and gray line), while RT points are plotted with a black outline. The RT sample without fill color does not fit the regression due to the formation of impurities during synthesis. Peaks are attributed to bond vibrations according to Sasaki et al.^30^ The locations of all peaks, statistics of the regressions, and plots of full spectra are presented in Table S4 and Figure S6. In panels E and F, missing RT points are outside the frame of the plot.

Since site vacancies in jarosite can occur at A- and B-sites, but not at T sites, we calculated element contents in jarosite relative to S, which is assumed to fill all T-sites in the crystal structures.^11^ In HT-Jrs, as the Al/(Al+Fe) molar ratio in the initial solutions increased from 0 to 0.4, the Al-for-Fe substitution (100 × Al/(Al+Fe)) in solids increased from 0.0 to 9.5% (Figure 3A and Table S3). Furthermore, the molar ratio (Al+Fe)/S decreased from 1.31 to 1.27 mol/mol S, and the K occupancy of the A-site increased from 0.40 to 0.44 mol/mol S (Figure 3A and Table S3). The Al content, relative to Fe, was expected to be lower in minerals than in the synthesis solutions because hydrolysis by Al^3+^ occurs at a higher pH than hydrolysis by Fe^3+^ (first hydrolysis constant, −logβ, of 4.6 to 5.0 and 2.19, respectively^45,46^).^24^ The differences in Fe and K occupancy from the theoretical 1.5 mol Fe/mol S and 0.5 mol K/mol S are explained by the B-site and A-site vacancy, respectively. Vacancies are commonly reported in the literature,^11,14,16^ being charge-balanced by protonation of OH^–^ groups elsewhere in the crystal structure.^47^ Aluminum was well mixed in 7.3% Al-substituted HT-Jrs and spatially correlated with Fe as seen in elemental distribution maps (Figure S2). Therefore, we calculated that the HT-Jrs samples ranged from K_0.80_(H_3_O)_0.20_Fe_2.62_(SO_4_)_2_(OH)_4.86_(H_2_O)_1.14_ to K_0.88_(H_3_O)_0.12_Fe_2.30_Al_0.24_(SO_4_)_2_(OH)_4.62_(H_2_O)_1.38_.

The elemental composition of HT-Jrs was quantified based on the EDX signal intensities extracted from elemental maps and integrated over the region marked in Figure S2A (full spectrum displayed in Figure S2), resulting in a K:Al:Fe:S ratio of 1.04:0.26:3.18:2, corresponding to Al-for-Fe substitution of 7.6% (Table S1). Regions that produced low signals of S or K (cf. region marked by red arrows in Figure S2B,D) indicate that this HT-Jrs contained a small amount of a phase other than jarosite. This may explain why the total K:S and Fe:S ratios derived from EDX measurements are above the theoretical stoichiometric maxima of 1:3 and 2:3, respectively.

Aluminum incorporation was lower in the RT-Jrs series than in HT-Jrs, since hydrolysis is less favored by Al^3+^ than Fe^3+^ at lower temperatures.^24^ When the Al/(Al+Fe) molar ratio was 0.4 in the initial solution, the Al-for-Fe substitution was 0.6% in the RT-Jrs product (Figure 3A and “RT 40%” in Table S3). The Al-for-Fe substitution increased to 8.0% with Al/(Al+Fe) in the synthesis solution of 0.6 (Figure 3A and “RT 60%” in Table S3) but Fe occupancy of the B-site exceeded the theoretical stoichiometric 3:2 limit of B:T ions, indicating that this ratio may be biased by the formation of other Fe or Al phases. RT-Jrs contained more pronounced Fe and K deficiencies than HT-Jrs, with K occupancy of the A-site ranging from 70% ± 2.3 to 56% ± 0.9 and Fe occupancy of the B-site as low as 72% ± 0.9 (Table S3 and Figure 3A). Trends in the amounts of A- and B- site deficiencies with increasing Al content were also less consistent in the RT-Jrs series. For samples with 0.0, 0.6, and 3.0% Al-for-Fe substitution, K deficiency was 30, 36, and 33%, respectively, and B-site deficiency was 25, 28, and 20%, respectively. Therefore, the minerals in 0.0 and 3.0% Al-for-Fe substituted RT-Jrs samples had formulas K_0.70_(H_3_O)_0.30_Fe_2.25_(SO_4_)_2_(OH)_3.75_(H_2_O)_2.25_ and K_0.67_(H_3_O)_0.33_Fe_2.33_Al_0.07_(SO_4_)_2_(OH)_5.20_(H_2_O)_1.80_, respectively. The Al-for-Fe substitution calculated based on EDX spectra of the unsubstituted RT-Jrs was 0.3% (Table S1), which likely represents the level of the instrumental background for Al. In the 0.6% Al-for-Fe-substituted RT-Jrs, the Al-for-Fe substitution derived from EDX measurements was 0.61% (Table S1). It is not possible to analyze the distribution of Al in the 0.6% Al-for-Fe substituted RT-Jrs sample (Figure S4), because the signal could not be separated from the instrumental background for Al.

In natural jarosite samples, the presence of other mineral phases has prevented precise measurements of Al substitution in jarosite from ASS in studies that used acid dissolution to determine the mineral composition.^22,23^ In acid dissolutions of our natural sample too, the theoretical maximum occupancy of the B-site in natural jarosite was apparently exceeded, with K: Fe: S stoichiometry of 0.86:3.07:2 and Al+Fe: S ratio of 3.33:2. Excess Fe and Al may be from clays or short-range ordered Al and Fe hydroxides that could not be physically separated from the natural jarosite sample. Rietveld fitting of XRD patterns indicated that the natural jarosite contained 92.6% jarosite and 7.84% quartz (Figure S10), but short-range ordered minerals may not have been detected by XRD. Organic carbon comprised 0.62% of the sample mass, and the HCl extraction of natural jarosite, which dissolved 89.3% of the sample mass, contained <5 mg/g of each of Si, P, Na, and Pb. Evidence for other phases, for example, clays or organically complexed Fe(III), was found in Mössbauer spectra of the natural jarosite in the form of a small doublet (2–6% of Fe in the sample) in spectra collected at low temperatures (Figure S15).^48,49^

We used the EDX spectra obtained from individual jarosite particles to improve the quantification of Al in the natural jarosite sample examined here. Aluminum was evenly distributed across the jarosite crystals. The elemental ratios were quantified in the two areas marked with blue and red squares in Figure 2A (spectra plotted in Figure S5). Assuming that all Al was incorporated into the jarosite crystal structure, Al-for-Fe substitution in these regions was 5.6 and 5.1%, respectively. The low Si signal intensity from these areas (c.f. Figure 2 F) and the strong correlations of Al with Fe, S, and K (c.f. Figure 2B–D) indicate that the Al is more likely associated with jarosite than silicate phases.

### 3.3 Unit Cell Dimensions

**Figure 4:**
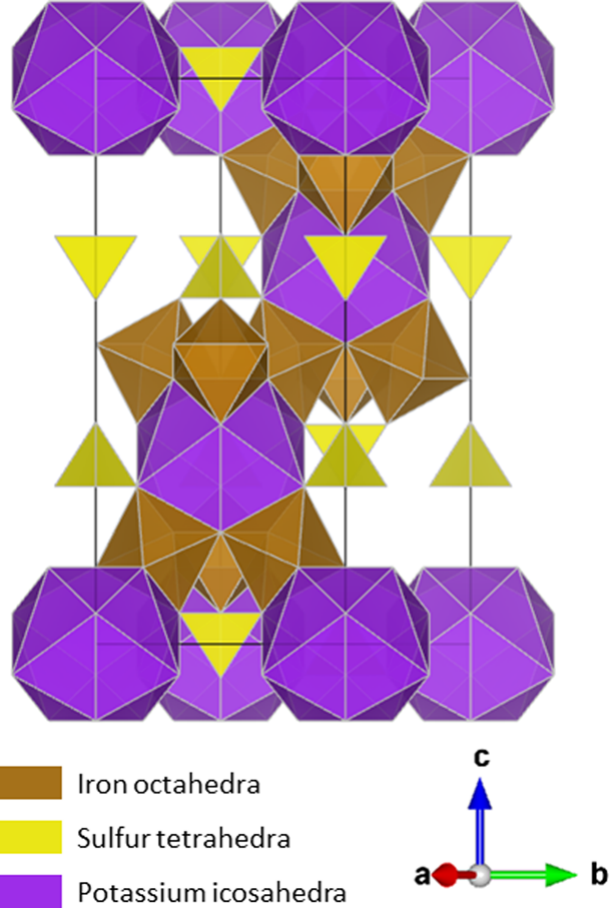
Visualization of a jarosite crystal.^40^ Yellow tetrahedra are SO_4_ groups centered on T sites, purple icosahedra are KO_20_ groups centered on A-sites, and brown octahedra are FeO_6_ groups centered on B-sites, which may be replaced with AlO_6_ groups, which are structurally equivalent but smaller. Black lines denote the border of the unit cell.

The unit cell dimensions of the jarosite were calculated by Rietveld fitting of XRD patterns. In HT-Jrs, the unit cell *a* dimension was linearly correlated with Al-for-Fe substitution (Al_sub_), consistent with substitution by Vegard’s law and previous characterizations of jarosite-alunite SSS.^13,24,26,27^ In this study, the unit cell dimensions follow the relationships *a*_0_ = 7.3141–0.0021 × Al_sub_ (*R*^2^ = 0.997) and *c*_0_ = 17.0550 + 0.0001 × Al_sub_ (*R*^2^ = 0.021), where *a*_0_ and *c*_0_ are the lengths of the *a* and *c* unit cell parameters in Å, respectively, and Al_sub_ is the percentage Al-for-Fe substitution [100 × Al/(Al+Fe)]. The relationships are plotted in Figure 3B,C, and the orientation of the crystal structure is visualized in Figure 4. The low *R*^2^ for the *c* parameter reflects the small systematic change caused by element substitution compared with the noise. RT-Jrs followed a trend similar to that observed in the HT-Jrs series, following the relationship *a*_0_ = −0.0020 × Al_sub_ + 7.316 (*R*^2^ = 0.87).

**Figure 5:**
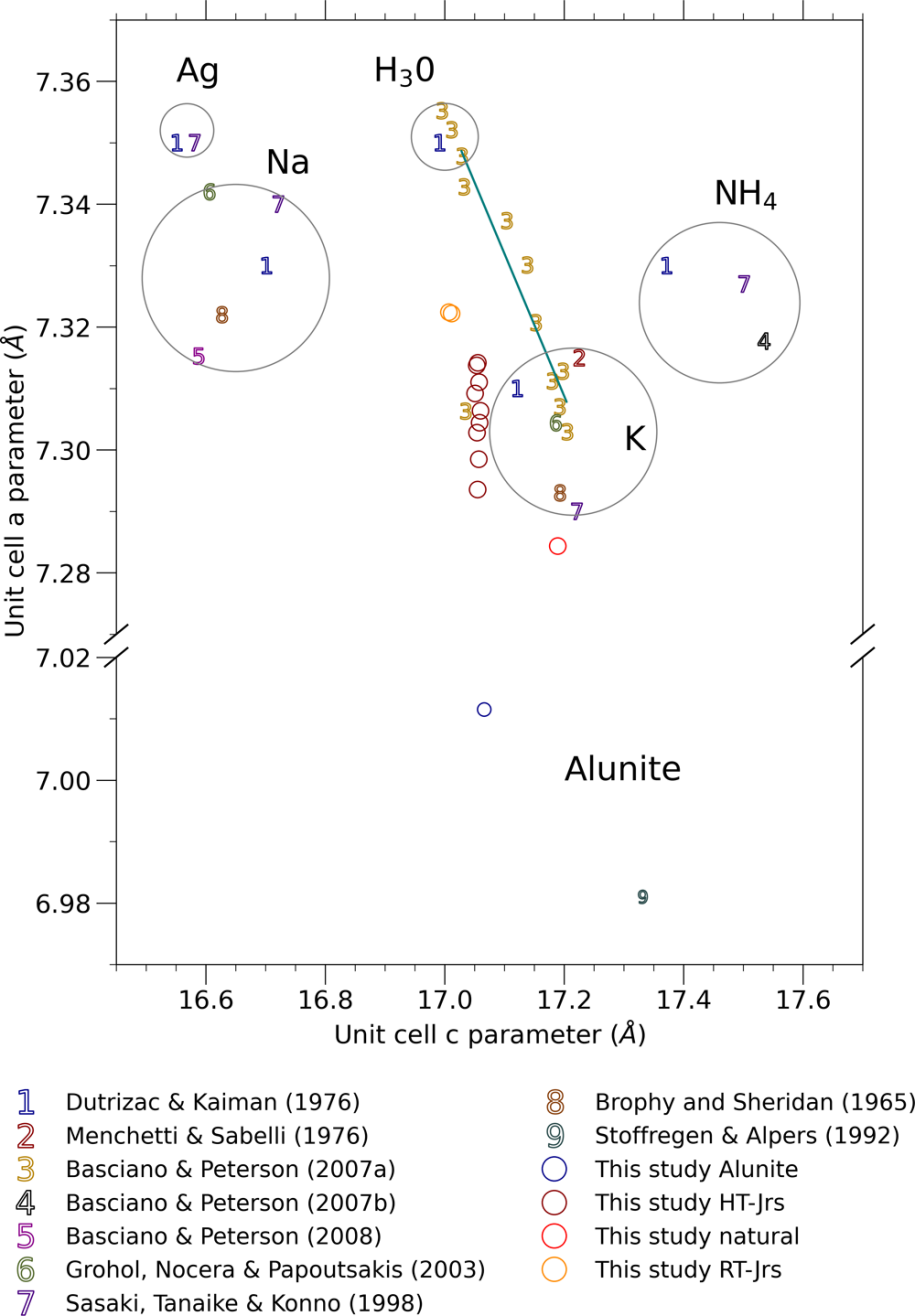
Plotted unit cell parameters of jarosite-alunite group minerals from the mineral samples analyzed in this study and selected previous studies.^30,40,50−53^ The regression, calculated from the SSS in Basciano and Peterson^50^ (plot marker 3), excludes the outlier at point (17.034, 7.306), which is Fe deficient.

Substitution at the B-site is well recognized as an influence on the *a* parameter,^24,26,27^ although A-site substitution also has an effect on the *a* parameter (Figure 5).^30,40,50−53^ For example, the K^+^-H_3_O^+^ jarosite series with full B-site occupancy follows the relationship *a*_0_ = 0.0006 × [H_3_O^+^] + 7.356, where [H_3_O^+^] is the H_3_O^+^-for-K substitution at the A-site in percent.^50^ The K^+^-H_3_O^+^ jarosite series is relevant here because K deficiency may be partially compensated by H_3_O^+^ substitution.^47,50^ Potassium deficiency in our HT-Jrs series is 13–20%, and therefore the *a* parameter is expected to be 0.008–0.012 Å larger (0.0006 Å per percent of H_3_O^+^ at the A-site) due to A-site H_3_O^+^ substitution. Even taking into account this effect of H_3_O^+^ substitution on the *a* parameter, increasing Al content in our HT-Jrs series was associated with a decrease in the length of the *a* dimension of the unit cell, corresponding to a change of 0.00046 Å per percent of Al substitution (from 7.3022 to 7.2859 Å; Figure 3B and Table S4). Linear regression of the deficiency-corrected *a* parameter of the HT-Jrs series produced the relationship *a*_0_ = −0.0018 × Al_sub_ + 7.3034 (*R*^2^ = 0.983).

The slope of our regression (−0.0018) is lower than that derived by Jones^13^ and lower than a regression that would include our alunite sample. However, using our regression to calculate the unit cell parameters of unsubstituted jarosite, we would expect an *a* parameter of 7.3034 ± 0.0010 Å (uncertainty is the 95% confidence interval of the regression), which is within the reported range of K-jarosite (Figure 5), near a theoretical K-jarosite on the regression through the jarosite series synthesized by Basciano and Peterson^50^ (7.2985 ± 0.0030 Å). Application of the correction factor for A-site K deficiency to RT-Jrs with Al-for-Fe substitution <3% produced an *a*-parameter estimation for unsubstituted jarosite of 7.2971 ± 0.0063 Å (uncertainty is the 95% confidence interval of the regression), which was also consistent with the corrected HT-Jrs series. The larger *a* parameter of RT-Jrs compared with HT-Jrs containing similar Al-for-Fe substitution (RT-Jrs sits above the regression through HT-Jrs samples in Figure 3B) could be caused by overestimated K occupancy of the A-site or Al substitution at the B-site of the RT-Jrs. The difference could be caused by a coexistent K- and/or Al-rich XRD-amorphous mineral phase that contributed to the total elemental content of the dissolved RT-Jrs sample. Although the unit cell parameters of jarosite may change as crystallinity increases,^54^ the similarity of the crystallite size of HT-Jrs and RT-Jrs indicates that crystallinity effects are not likely to be the cause of the observed difference in the *a* parameter.

The use of XRD-derived unit cell dimensions to quantify unknown levels of Al substitution in samples of natural jarosite is limited by the potential influence of multiple substituting ions and uncertainty in the quantification of standard minerals. In our natural jarosite, the *a* parameter of the unit cell (7.2844 Å) is smaller than the *a* parameter of any synthetic jarosite analyzed here, or reported in previous studies (Figure 5),^30,40,50−53^ except for minerals in the jarosite-alunite solid solution series.^24,26,27^ Substitution for Fe^3+^ with ions smaller than Fe^3+^, such as Al^3+^, is a dominant cause of a smaller *a* parameter. Increasing the occupancy of the A-site with H_3_O^+^, Na^+,^ and NH_4_^+^, as well as rarer ions in ASS such as Pb^2+^ and Ag^+^, would increase the size of the *a* parameter. Similarly, substitution for SO_4_^2–^ by AsO_4_^3–^ increases both the *a* and *c* parameters.^16,55^ Therefore, we calculate that the Al occupancy of the natural jarosite is at least 8 to 9% (based on our regression or that from ref (13)), which is somewhat higher than the estimation from the analysis of element maps (5.1–5.6%).

The estimate of Al substitution in the natural jarosite could be altered if the substitution for K at the A-site was compensated. The occupancy of the A-site is linked to the size of the unit cell *c* parameter,^30,40,50−53^ and our data confirm that B-site substitution does not affect the *c* parameter.^13,24^ The measured *c* parameter of the natural jarosite (17.1894 Å) is close to K-jarosite.^30,40,50^ Notwithstanding the effect of B-site vacancies^50,56^ and the effect of small amounts of H_3_O^+^, Na^+^, and Pb^2+^ substitutions, the c parameter of the natural jarosite is consistent with abundant K in the dissolved sample relative to other ions that may occupy the A-site.

The unit cell dimensions of alunite-jarosite minerals are a robust indicator of elemental substitution. The effect of various substitutions on the unit cell dimensions has been well characterized, allowing for the analysis of structural substitutions with high confidence. Although resolving multiple substitutions with the two unit cell dimensions of jarosite is an underdetermined problem, we demonstrated here that the analysis of the unit cell parameters of jarosite can be useful to deduce information about the substitutions that occur in a natural jarosite sample.

### 3.4 Bond Vibration

A consistent relationship was observed between the Al occupancy of the B-site in the HT-Jrs and the position of Raman spectral peaks (Table S5, Figures 3D–H and S11). However, changes in occupancy at the A-site could also produce differences in the Fe–O and SO_4_ vibrational bands.^29−31^ It was previously shown that strong negative correlations exist between the unit cell *c* parameter (caused by A-site substitution) and peak positions near 223, 358, 434, 1007, and 1102 cm^–1^ [O–B(1), OH–B_(3)_, ν_2_(SO_4_), ν_1_(SO_4_), and ν_3_(SO_4_), respectively,^32^ where B is Fe or Al, depending on the sample].^30^ By contrast, no strong correlations were observed for peaks near 301, 454, and 625 for [O–B_(2)_, O/OH–B_(4)_, and ν_4_(SO_4_), respectively^32^].^30^ Therefore, in our HT-Jrs series, the increasing wavenumber of the O–B_(1)_, O–B_(3)_, and ν_1_(SO_4_) peaks may be related to the effect of H_3_O^+^-for-K substitution on the *c* parameter, with unknown contribution from the *a* parameter. However, strong correlations were observed between increasing Al content in our HT-Jrs series and increasing wavenumber of the ν_4_(SO_4_), O–B_(2)_, and O/OH–B_(4)_ peaks. Therefore, these peaks were probably influenced by changes in the *a* parameter due to Al-for-Fe substitution without interference from substitution at the A-site. Among these three vibration bands, the largest change in the peak position across the HT-Jrs series was observed in the ν_4_(SO_4_) peak (0.196 cm^–1^ per percent Al; Table S5). Both the positions of the O–B_(2)_ and the O/OH–B_(4)_ demonstrate good correlations (*R*^2^ = 0.95 and Table S5), but the position of the O/OH–B_(4)_ lies close to a cluster of peaks, which may increase uncertainty in the fit.

Peak positions of the RT-Jrs series showed some deviation from the trends observed in HT-Jrs with similar levels of Al-for-Fe substitution. B-site deficiency, which is much higher in the RT-Jrs than HT-Jrs, and not well correlated to the Al content, could contribute to the location of the ν_4_(SO_4_) and O–B_(2)_ peak locations. Since peak positions are related to the unit cell parameters, greater B-site deficiency could cause higher Raman peak wavenumbers. In high-Al RT-Jrs samples with Al-for-Fe substitution >3%, a peak at 713 cm^–1^ confirms the co-occurrence of another mineral phase, consistent with the XRD-amorphous phase mentioned in the previous section, which could not be matched with any spectra in available Raman spectroscopy databases (PODERA5, POTERA5, SUPHRA5, INOFRA5, INOPRA5, MINFRA5, MINPRA5, S.T. Japan-Europe Gmbh, 2017; RRUFF, RRUFF project, USA; Minerals and inorganic materials and polymer database, Renishaw plc, UK). The impurities could be any of several poorly characterized short-range ordered Al hydroxysulfate minerals that are known to form in acid sulfate environments.^1^ Several Raman spectral peak locations in these RT-Jrs samples are likely influenced by the phase impurities.

When applied to natural jarosite, the measurement of Raman signals is hindered by the fluorescence of co-occurring clays and/or organic matter. The baseline subtraction removes the fluorescence signal, but the resulting Raman spectra are noisier than spectra of synthetic minerals (as seen in Figure S11), and the peak location may not be determined as precisely. The position of the ν_4_(SO_4_) peak of natural jarosite falls within the range of the HT-Jrs series, between the 3.0 and 3.9% Al-for-Fe substituted samples. However, the position of the O–B_(2)_ peak is above the range of the HT-Jrs series (corresponding to Al-for-Fe substitution of 16%) and the RT-Jrs series. The location of the O–B_(2)_ peak may be biased by the location of a nearby goethite peak (299 cm^–1^, or higher with Al substitution).^57^ Because of the common occurrence of goethite with jarosite in ASS, the O–B_(2)_ peak may not be appropriate for characterizing natural jarosite from such environments. The position of the O/OH-B_(4)_ falls below the range of the HT-Jrs and RT-Jrs series, probably because of uncertainty fitting this peak in the noisy spectrum. The peak locations that are strongly correlated with the *c* parameter [O–B_(1)_, OH–B_(3)_, ν_2_(SO_4_), ν_1_(SO_4_)] differed for the HT-Jrs series and natural jarosite because of differences in K occupancy. While the O–B_(1)_ and ν_2_(SO_4_) positions of natural jarosite fall outside the range of HT-Jrs series, the positions of the OH–B_(3)_ and ν_1_(SO_4_) are similar. This may indicate that OH–B_(3)_ and ν_1_(SO_4_) are affected by both the *a* and *c* parameters of the unit cell.

Raman spectroscopy is a useful tool in certain situations, such as for the identification of poorly crystalline minerals,^29^ spatially resolved measurements,^58^ and in situ measurements, for example, on Mars.^59^ However, interference from the goethite spectrum and fluorescence present challenges to the application of this method to quantify Al-for-Fe substitution in natural jarosite. An improved holistic understanding of the effects of multiple substitutions on each Raman spectral peak would improve the application of this method, by allowing many peaks to be analyzed simultaneously.

### 3.5 Effect of Al-for-Fe Substitution on the Hyperfine Structure

B-site substitution in jarosite-alunite minerals alters the interaction of Fe atoms in the jarosite crystal. As a result, Al-for-Fe substitution in jarosite has been observed to increase quadrupole splitting (QS) in Mössbauer spectra at room temperature, from 1.15 ± 0.05 to 1.25 ± 0.05 mm/s when Al-for-Fe substitution increased from 0 to 17%^33^ and from 1.15 to 1.30 mm/s (no uncertainty reported) when Al-for-Fe substitution increased from 0 to 83%.^35^ In HT-Jrs with 7.3% Al-for-Fe, QS increased from 1.123 ± 0.002 to 1.136 ± 0.008 mm/s at 77 Kand quadrupole shift (ε) from −0.081 ± 0.004 to −0.093 ± 0.009 mm/s at 5 K (Table 1, Figures S12 and S13; reported error is 2SD derived from the covariance matrix). The change in QS or ε in the RT-Jrs series (Table 1 and Figure S13) was too small to be interpreted. While the change in QS in our HT-Jrs samples was consistent with earlier estimations,^33,35^ the uncertainty estimation is derived from the covariance matrix for a single sample, and such small changes of the QS or ε in jarosite with Al-for-Fe substitution below 7% (as considered here) may be difficult to interpret when additional sources of uncertainty are considered. Absolute differences between the QS and ε recorded in K-jarosite and samples in this study may be attributed to H_3_O^+^ substitution at the A-site of HT-Jrs (decreased *c* parameter).^34^ Furthermore, increasing B-site vacancy through the HT-Jrs series may have contributed to increased QS or ε.^34,35^ A stronger effect was observed in the hyperfine field (*H*) at 5 K. Aluminum-for-Fe substitution of 7.3% corresponded to a decrease in the *H* from 47.56 ± 0.04 to 46.85 ± 0.13 T.

**Table 1:** Fitted Mössbauer Parameters at 4.2–5 and 77 K for Selected Hydrothermal (HT), Room Temperature (RT), and Natural Jarosite Samples^a^

sample name	temp.	reduced χ^2^	HWHM	comp area (%)^b^	CS (mm/s)	QS or ε (mm/s)	σ_QS_ or σ_ε_ (mm/s)	*H* (T)	σ_H_ (T)	site prop. (%)^c^
HT, No Al sub	77 K	11.17	0.135*	100	0.48396(88)	1.1230(17)	0.2890(22)			
	4.2 K	4.05	0.135*	100	0.4891(35)	–0.0810(35)	0*	47.559(44)	1.304(99)	65.2618*
								43.80(88)	3.27(44)	34.7(76)
HT, 1.1% Al sub	77 K	1.20	0.135*	100	0.4821(36)	1.1253(69)	0.2770(91)			
	4.2 K	1.92	0.135*	100	0.4847(59)	–0.0918(59)	0*	47.262(76)	1.49(15)	64.5908*
								43.3(12)	3.86(57)	35.4(95)
HT 3.4% Al sub	77 K	6.74	0.135*	100	0.4874(11)	1.1219(22)	0.2819(28)			
	5 K	7.05	0.135*	100	0.4931(24)	–0.0921(24)	0*	47.198(34)	1.512(61)	58.5642*
								43.60(37)	3.96(15)	41.4(33)
HT 7.3% Al sub	77 K	1.05	0.135*	100	0.4809(41)	1.1358(79)	0.276(10)			
	4.2 K	1.22	0.135*	100	0.4846(93)	–0.0927(92)	0*	46.85(13)	1.45(22)	45.312*
								43.88(70)	4.41(35)	54.7(79)
RT, No Al sub	77 K	15.87	0.135*	100	0.48696(80)	1.1005(15)	0.3090(19)			
	5 K	15.01	0.135*	100	0.4900(15)	–0.0832(15)	0*	47.583(18)	1.267(40)	62.6849*
								43.81(31)	3.37(16)	37.3(28)
RT, 0.6% Al sub	77 K	8.35	0.135*	100	0.4860(13)	1.0714(24)	0.3364(29)			
	5 K	9.61	0.135*	100	0.4908(20)	–0.0882(20)	0*	47.050(32)	1.444(63)	57.0085*
								43.75(35)	3.40(15)	43.0(40)
Natural^d^	77 K 1st var.^e^	0.90	0.135*	67.8(29)	0.48405(100)	1.2869(45)	0.1658(91)			
				23.7(31)	0.484282*	0.972(58)	0.437(23)			
				8.54(75)	0.460(28)	–0.115(28)	0*	47.91(24)	1.92(28)	
	77 K 2nd var.^e^	1.30	0.135*	84.96(61)	0.48319(90)	1.2676(20)	0.1992(30)			
				7.36(34)	0.45*	0.46*	0.27*			
				7.68(57)	0.455(28)	–0.114(28)	0*	47.97(24)	1.67(28)	
	5 K	22.43	0.135*	1.39(15)	0.399(23)	0.470(42)	0*			
				88.48(54)	0.4808(13)	–0.0643(13)	0*	48.386(20)	0.818(49)	64.7187*
								46.14(31)	2.17(14)	35.3(44)
				10.14(53)	0.6332(59)	–0.1958(57)	0*	49.456(54)	0.19(15)	

a Parameters that were fixed during modeling are marked with an asterisk. The uncertainty (2SD) of the final digit is expressed in parentheses based on the covariance matrix of the fit. Fitted parameters were center shift (CS), quadrupole splitting (doublets; QS) or quadrupole shift (sextets; ε), the standard deviation of QS or ε (σ_QS_ or σ_ε_, respectively), hyperfine field (*H*), and standard deviation of the hyperfine field (σ_H_).

b The relative area of modeled components and the relative ratio of sites modeled as part of the same component.

c The relative area of modeled components are listed as Comp Area and Site Prop.

d The natural jarosite sample contains phases that can be attributed to minerals other than jarosite.

e Two alternative fits of the natural jarosite spectrum at 77 K are presented, and the differences are discussed in the main text.

In the natural jarosite, the ε and H of the spectrum collected at 5 K were higher than those of any HT-Jrs sample, consistent with greater B-site Fe occupancy. Interference between jarosite and Fe oxyhydroxides, such as goethite, may be a limiting factor when estimating the precise QS and H of jarosite in natural samples. At 77 K, the dominant part of the spectrum is not satisfactorily modeled with a single doublet, and we present two approaches to analyze this spectrum. The first (“1st var.” in Table 1) was similar to the approach taken for another natural jarosite sample from ASS, whereby two doublet components were fit and both attributed to jarosite.^60^ The QS values of the two doublets that fit in our natural jarosite were above and below the QS of the HT-Jrs series. However, we consider it likely that the measured spectrum contains an Fe(III) doublet caused by Fe(III) phases such as phyllosilicates or organically complexed Fe(III).^48,49^ We observe such a doublet in spectra below the temperature of superparamagnetic relaxation, otherwise known as the blocking temperature, *T*_B_ (1–8% of the spectral area) and in soil measured at the same location. The Fe(III) phase could not be fit with floating parameters in jarosite samples at 77 K, but typical parameters for this phase were chosen based on measurements of soil (“2nd var.” in Table 1) and the remainder was fit as a single doublet. The parameters of the dominant phase resembled the HT-Jrs. The higher QS in the natural jarosite (QS = 1.2676 in fit “2nd var.”; Table 1) than in HT-Jrs was consistent with high B-site Fe occupancy.

The subtle responses of QS, ε, and H in Mössbauer spectroscopy to Al-for-Fe substitution in jarosite may limit the interpretation of these parameters to identify and quantify Al-substitution in jarosite in many situations. However, the robustness of the typical parameters for jarosite is useful when fitting Mössbauer spectra that contain jarosite with unknown B-site occupancy.

### 3.6 Magnetic Ordering

**Figure 6:**
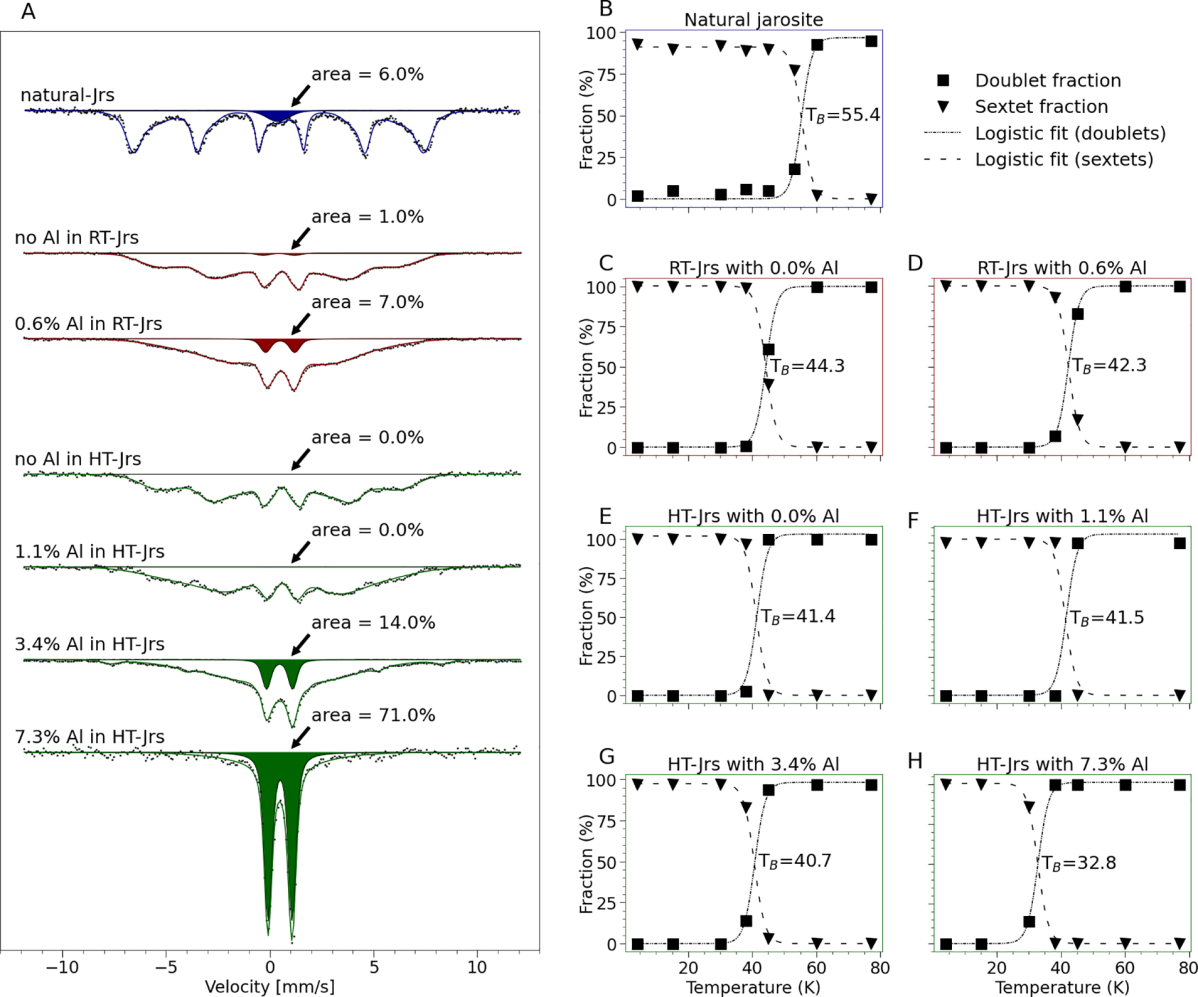
(A) Plot of Mössbauer spectra of natural jarosite and jarosite samples from RT-Jrs and HT-Jrs series, each measured at 38 K. Black dots indicate measured points, colored lines are the sum of fitted components, and the colored region is the fitted paramagnetic component, which was consistent with jarosite above its paramagnetic blocking temperature, *T*_B_, for all samples except natural jarosite, which contained a paramagnetic nonjarosite phase. (B–H) Mössbauer fitting results provide information on the *T*_B_. Each plot shows the relative size of the doublet and sextet components of the spectra measured at a series of temperatures above and below *T*_B_. The data points (plotted as unconnected points) were fit with logistic functions (plotted as lines) using “scipy optimize”,^65^ with fixed logistic growth rate, *k* = 0.7, for all curves. The domain value (temperature) at the intersection of the curves for the sextet and doublet fractions is taken as *T*_B_ and annotated on each plot. All measured spectra are available in Figures S12–S15.

Previous investigations of jarosite-alunite group minerals with Mössbauer spectroscopy have not systematically considered the effect of Al substitution on the *T*_B_, defined as the temperature at which 50% of the mineral is magnetically ordered^61^). In goethite, Al substitution alters the mineral's magnetic characteristics, and a relationship between *T*_B_, crystallite size, and degree of Al substitution can be derived.^62,63^ One previous study of 17% Al-for-Fe substituted K-jarosite found a *T*_B_ between 45.7 and 48.6 K.^64^ In this study, we collected spectra at a series of temperatures around the blocking temperatures of selected samples (Figures S12–S15) and fit the relative area of the magnetically ordered sextet and paramagnetically ordered doublet (Figure 6B–H). While the *T*_B_ of unsubstituted HT-Jrs was between 38 and 45 K (*T*_B_ of ca. 41.4 K; Figure 6E), the *T*_B_ of 7.3% Al-for-Fe substituted HT-Jrs was between 30 and 38 K (*T*_B_ of ca. 32.8 K; Figure 6H). Smaller crystallite sizes, which corresponded to higher Al content in HT-Jrs (Table S4), may have also contributed to lower *T*_B_. The RT-Jrs series exhibited a slightly higher *T*_B_ than HT-Jrs, but it was also ordered over a greater range of temperatures. For example, the 1.1%-Al-for-Fe substituted HT-Jrs had *T*_B_ of ca. 41.5 K (Figure 6), whereas the 0.5% Al-for-Fe substituted RT-Jrs had *T*_B_ of ca. 42.3 K (Figure 6D). The effects of Al substitution on *T*_B_ can be illustrated by using the spectra collected at 38 K (Figure 6A). At this critical temperature, a larger magnetically ordered sextet fraction of the jarosite phase was associated with a higher *T*_B_.

The natural jarosite spectra (Figure S15) comprised three fitted phases: One that was magnetically ordered at all temperatures, one that was not magnetically ordered at any temperature, and a phase (the major component, attributed to jarosite) that underwent magnetic ordering between 53 and 60 K. Although *T*_B_ of natural jarosite was higher than both RT-Jrs and HT-Jrs, the LVol-IB of natural jarosite measured by XRD (83 nm) was within the range of the HT-Jrs series. Therefore, crystallite size is not the most likely cause of the higher *T*_B_. Greater total B-site occupancy of natural jarosite than the HT- and RT-Jrs series is a plausible explanation.

While higher Al substitution was correlated with lower *T*_B_ in each series, both the crystallite size and total B-site cation occupancy can also affect *T*_B_. Aluminum occupancy differences greater than approximately 3–5% can be interpreted, even when measured on samples containing multiple Fe phases. However, Al occupancy should not be quantified in samples in which defects and crystallite sizes may also affect *T*_B_ until those effects are quantified.

## 4 Conclusions

Aluminum-for-Fe substitution in jarosite can be identified and quantified by XRD, Raman spectroscopy, and Mössbauer spectroscopy in bulk samples and by TEM-EDX on individual crystals. Each of these methods provides a means of characterizing the chemical composition of jarosite when mixed with other minerals such as Al-rich clays. Strong correlations were observed between the Al content and *c* parameter, which could be measured by XRD, allowing for confident predictions of Al-for-Fe substitution. Strong correlations were also observed between Al content and ν_4_(SO_4_) and O–B_(2)_ Raman peak locations, although interferences from other minerals that are associated with natural jarosite may limit the interpretation of these peaks. In Mössbauer spectroscopy, the QS, ε, and H had subtle responses to substitution, demonstrating the robustness of the typical parameters for recognition of jarosite minerals with unknown B-site occupancy. However, *T*_B_ may provide valuable information about the B-site occupancy of jarosite samples with a similar crystallinity. In the natural jarosite, elemental distribution maps of Al, K, Fe, S, and Si provided direct evidence for Al-for-Fe substitution of approximately 5% in jarosite from ASS, which was consistent with the small *a* parameter of the unit cell in comparison with K-Jrs references and the correspondence between the resulting O–Fe_(2)_ Raman peak positions of natural and Al-substituted HT-Jrs. The elevated *T*_B_ and higher *c* parameters, compared with the HT-Jrs and RT-Jrs, indicated high levels of B-site occupancy in the natural jarosite sample. Therefore, we propose that the composition of natural jarosite was approximately K(Fe_2.85_Al_0.15_)(SO_4_)_2_(OH)_6_.

Quantification of elemental composition of jarosite using the techniques employed here can offer new insight into the characteristics of natural jarosite and therefore new information regarding the environments in which it is found. The strengths and weaknesses of each technique lend them to different applications. However, when compared, their complementarities may reveal the effects of multiple substitutions. Challenges to quantification in some circumstances remain because of a lack of information about some SSS in the alunite group, including minerals with B-site vacancies. However, this study provides previously unavailable information regarding the effect of B-site substitution, including changes in parameters that had previously been identified with A- and T-site substitution, and lays the foundation for improved characterization of jarosite-alunite minerals that occur in the environment.

## Supporting Information Available

Soil photograph; additional EM images, EDX spectra, and EDX analysis; elemental analysis results; XRD patterns and fits; Raman spectra and data analysis; Mössbauer spectra and fits (PDF)
