# Supplementary material for: Structural Effects of Aluminum and Iron Occupancy in Minerals of the Jarosite-Alunite Solid Solution
Source: ACS Earth Space Chem. 2024 Jan 25;8(2):194–206. doi: 10.1021/acsearthspacechem.3c00174 (PMC10875661; doi:10.1021/acsearthspacechem.3c00174)
Supplement: Supplementary file 1 — sp3c00174_si_001.pdf [file sp3c00174_si_001.pdf]

Supplementary Material for

Structural effects of aluminum and iron occupancy in minerals of the jarosite-alunite solid solution

Andrew R. C. Grigg[a], Luiza Notini[a], Ralf Kaegi[b], Laurel K. ThomasArrigo[a,c], Ruben Kretzschmar[a]

[a] Soil Chemistry Group, Institute of Biogeochemistry and Pollutant Dynamics, Department of Environmental Systems Science, ETH Zurich, Universitätstrasse 16, CHN, CH-8092 Zurich, Switzerland

[b] Eawag, Swiss Federal Institute of Aquatic Science and Technology, Überlandstrasse 133, CH-8600 Dübendorf, Switzerland

[c] Environmental Chemistry Group, Institute of Chemistry, Avenue de Bellevaux 51, University of Neuchâtel, CH-2000 Neuchâtel, Switzerland

25 Pages, 15 Figures, 6 Tables

Contents

1 Soil profile ... 2
2 Electron microscopy ... 3
3 Mineral colours ... 9
4 Elemental analysis ... 10
5 X-ray diffraction ... 12
6 Raman spectroscopy ... 15
7 Mössbauer spectroscopy ... 17
8 References ... 25

1 Soil profile

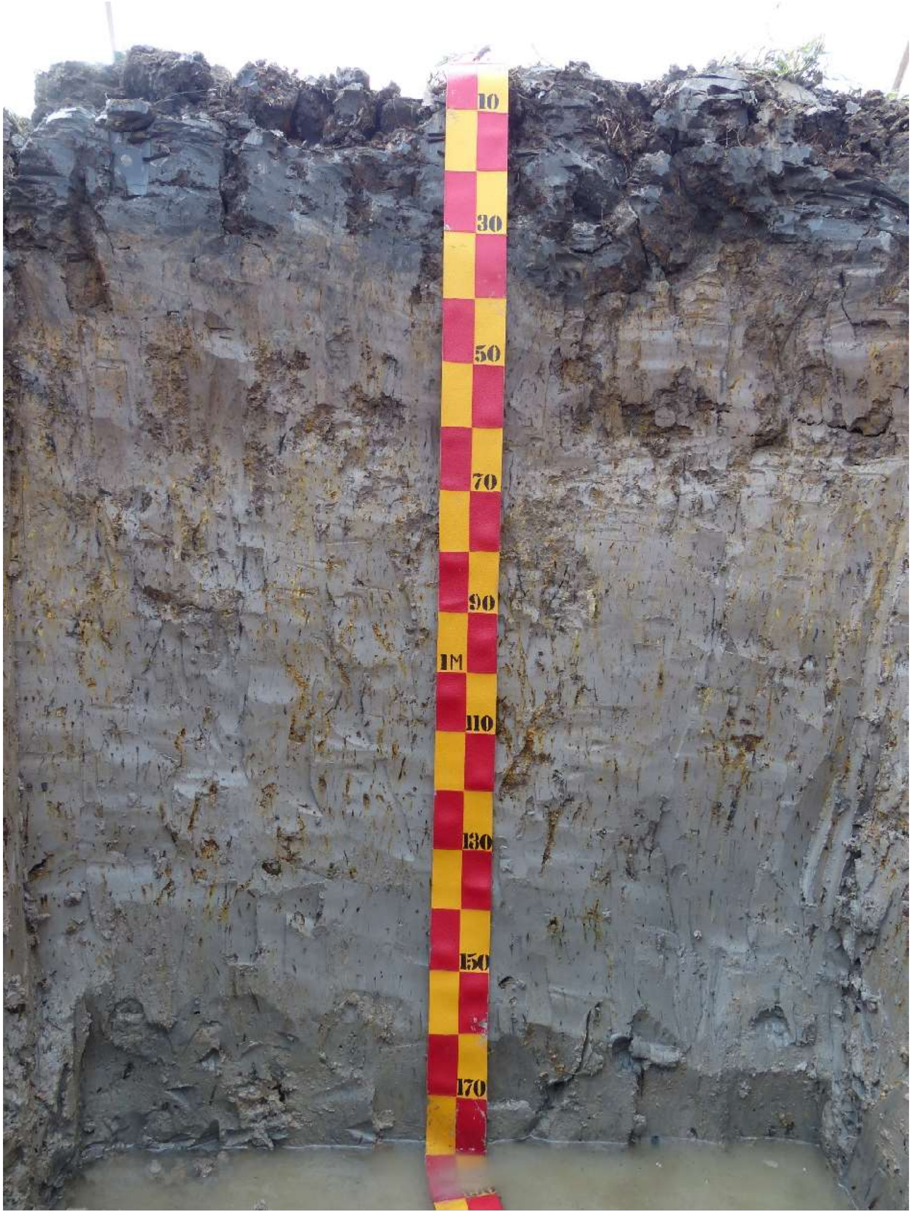

Figure S1: Soil profile from which the natural jarosite sample was collected. Jarosite can be seen in the profile as yellow flecks between 68 cm and 135 cm depth.

2 Electron microscopy

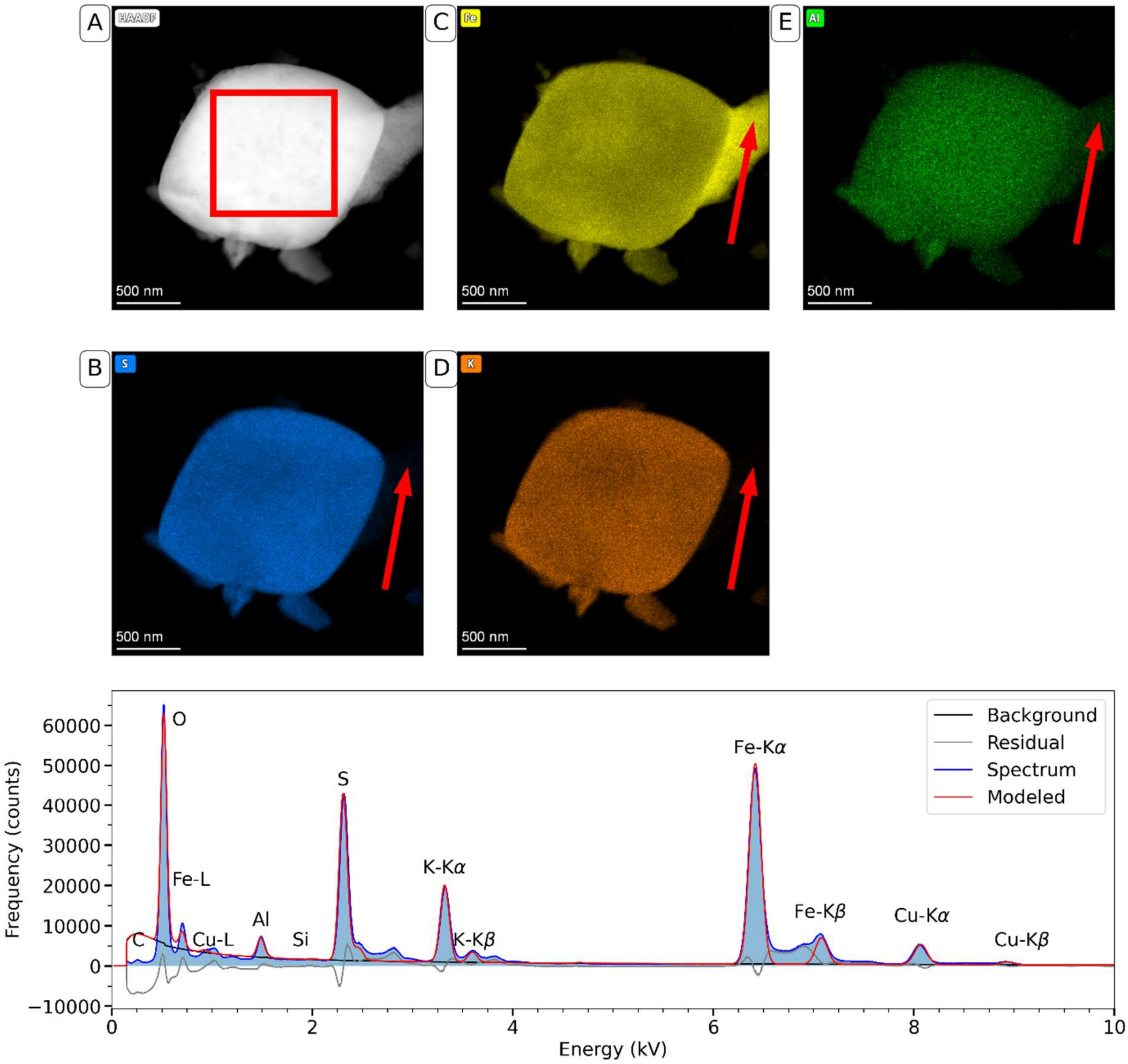

Figure S2: Elemental distribution maps derived from EDX analyses of a sample of 7.3% Al-substituted hydrothermally-synthesised jarosite (HT-Jrs). Panels A-E show the high angle annular dark field image (HAADF), distribution of S, distribution of Fe, distribution of Al and distribution of K respectively. The spectrum in the bottom panel represents to the integrated signal intensities from the area marked by the red box in panel A. The red arrows in Panels B to D indicate the location of a particle that produced a signal in the HAADF image but had low S and K content, as discussed in the main text.

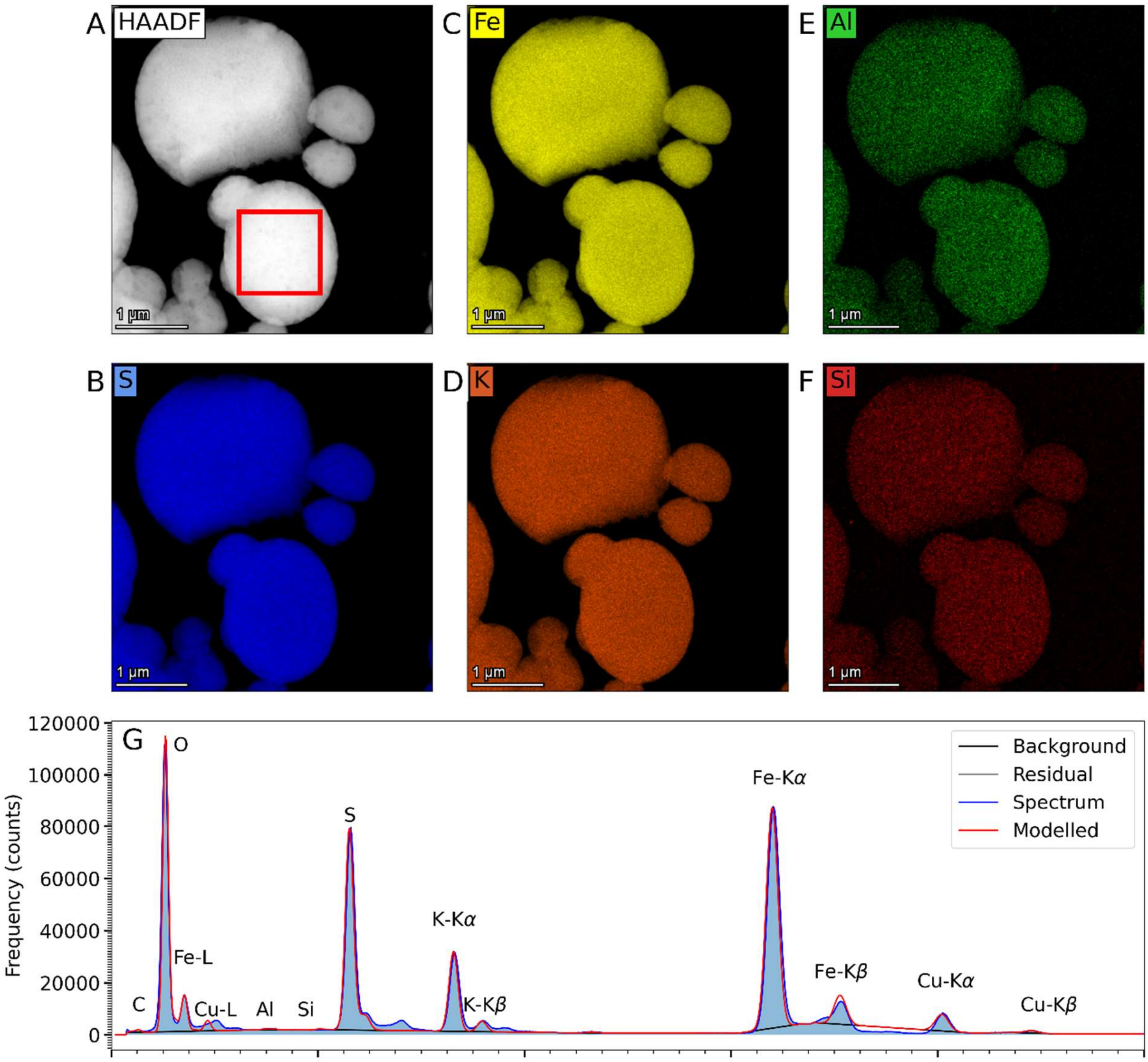

Figure S3: Elemental distribution maps of unsubstituted room-temperature-synthesised jarosite (RT-Jrs). Panels A-E show the high angle annular dark field image (HAADF), distribution of S, distribution of Fe, distribution of Al and distribution of K and distribution of Si, respectively. The spectrum in the bottom panel represents to the integrated signal intensities from the area marked by the red box in panel A, from which elemental ratios were calculated (Table S3).

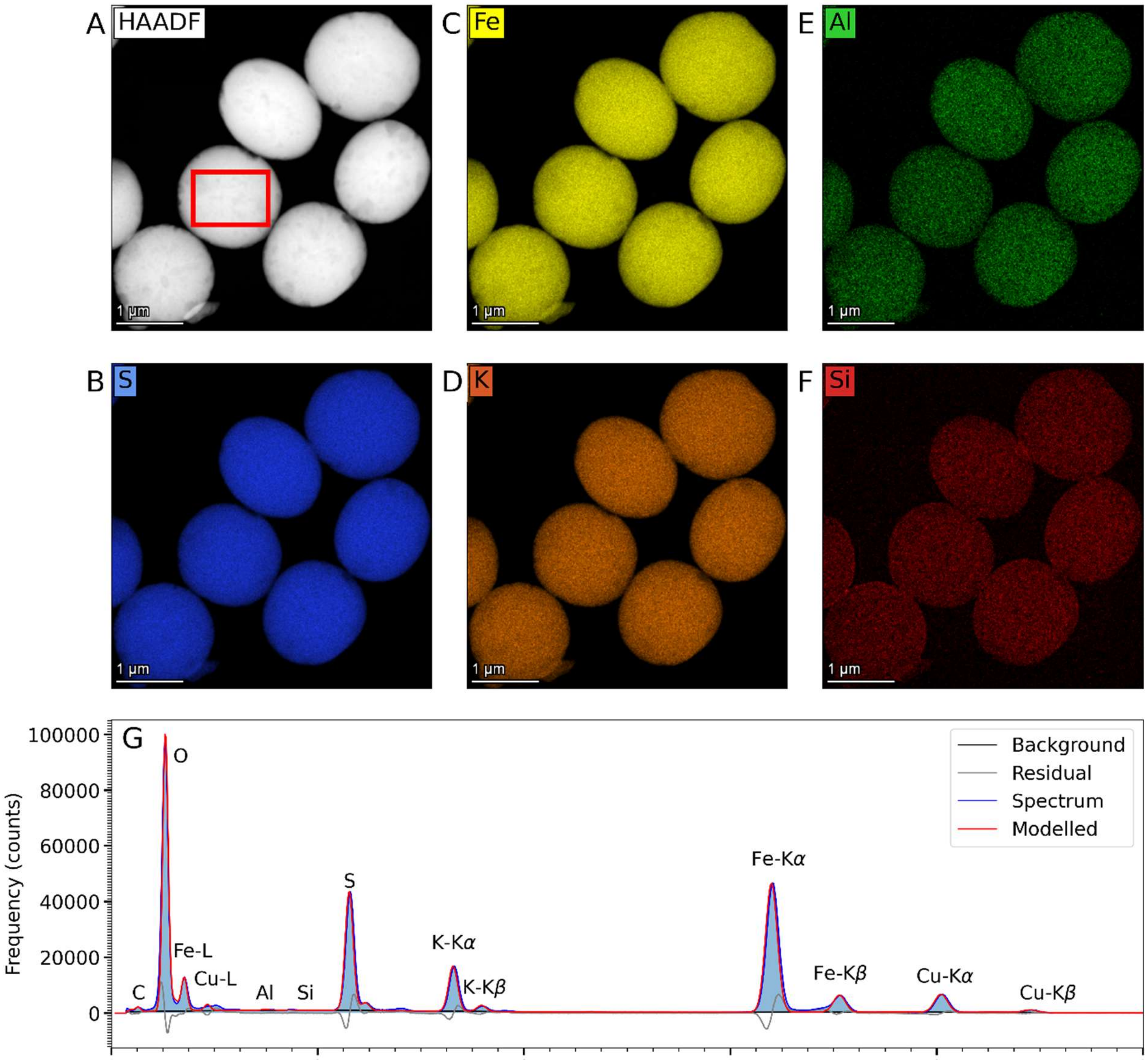

Figure S4: Elemental distribution maps of a sample of 0.6%-Al-for-Fe-substituted room-temperature-synthesised jarosite (RT-Jrs). Panels A-E show the high angle annular dark field image (HAADF), distribution of S, distribution of Fe, distribution of Al and distribution of K and distribution of Si, respectively. The spectrum in the bottom panel represents to the integrated signal intensities from the area marked by the red box in panel A, from which elemental ratios were calculated (Table S3).

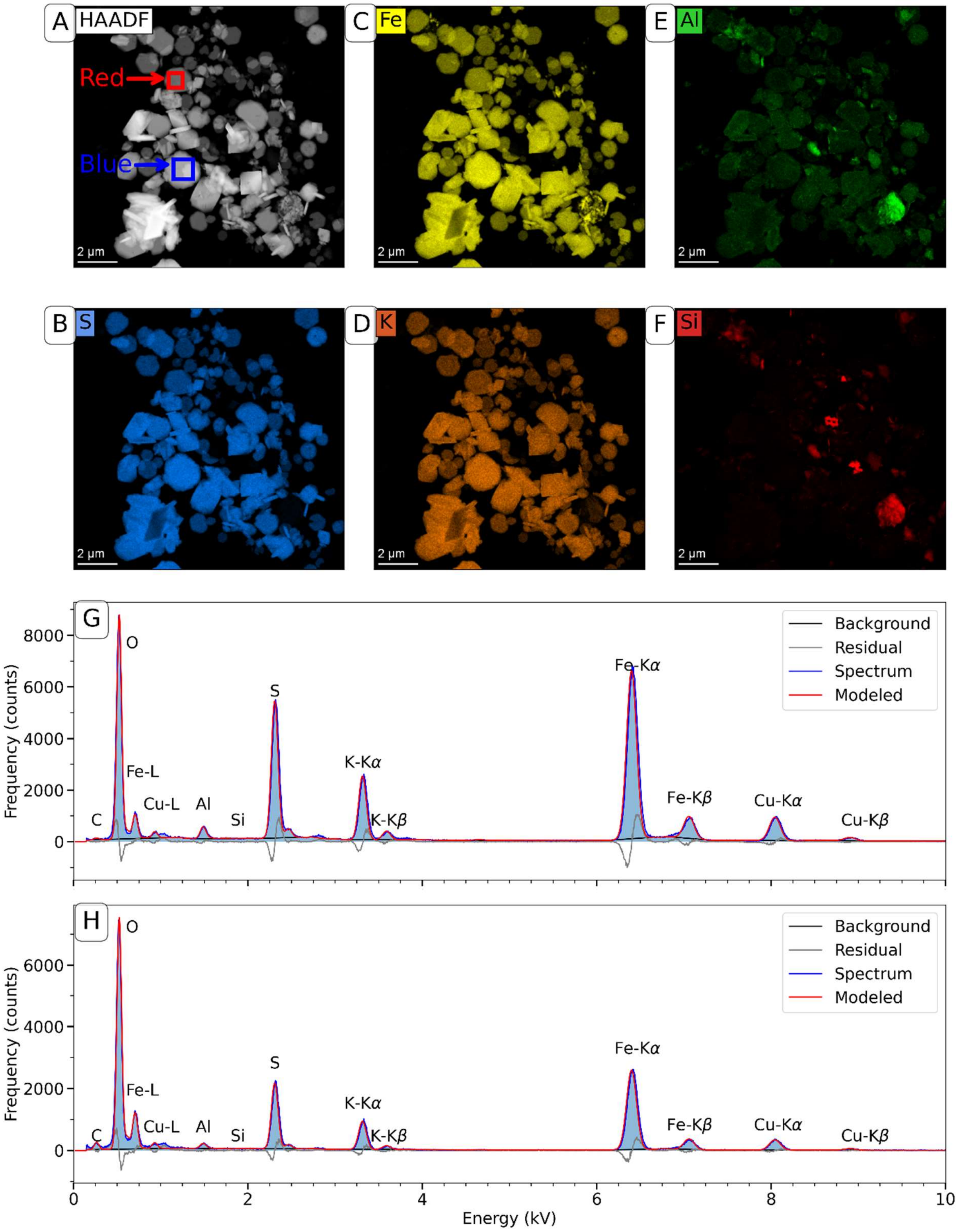

Figure S5: Elemental distribution maps of a sample of natural jarosite from Thailand. Panels A-F: High angular dark-field image (HAADF), distribution of S, distribution of Fe, distribution of K, distribution of Al and distribution of Si, respectively, reproduced from Figure 2. Panels G & H: The EDX spectra extracted from the areas indicated by red and blue boxes in Panel A.

Table S1: Elemental composition of individual jarosite particles derived from EDX measurements in areas marked by coloured squares in Figures S2A, S3A, S4A and S5a. [a] Elemental content presented as normalised atom percent abundance. [b] S-norm refers to the abundance of key elements in the jarosite structure, normalised to the expected molar ratio of S in jarosite (2 mol/mol). [c] The Al-for-Fe substitution is calculated as the content of Al normalised to the sum of Al and Fe and presented as a percentage (100×Fe/(Al+Fe)).

	7.3% Al-for-Fe HT-Jrs (Fig S2)			Unsubstituted RT-Jrs (Fig S3)			0.6% Al-for-Fe RT-Jrs (Fig S4)			Natural jarosite (Fig 2 & Fig S5, red)			Natural jarosite (Fig 2 & Fig S5, blue)		
Element	Norm. abundance [atom %] [a]	S-norm. [b]	Al-for-Fe substitution [atom %] [c]	Norm. abundance [atom %] [a]	S-norm. [b]	Al-for-Fe substitution [atom %] [c]	Norm. Abundance [atom %] [a]	S-norm. [b]	Al-for-Fe substitution [atom %] [c]	Norm. abundance [atom %] [a]	S-norm. [b]	Al-for-Fe substitution [atom %] [c]	Norm. abundance [atom %] [a]	S-norm. [b]	Al-for-Fe substitution [atom %] [c]
O	42.43	4.79		45.68	4.93		56.76	7.19		44.79	5.06		64.28	7.26	
Al	2.34	0.26	7.67	0.09	0.01	0.33	0.13	0.02	0.61	1.59	0.18	5.61	0.93	0.11	5.06
S	17.71	2 [b]		18.55	2 [b]		15.77	2 [b]		18.08	2 [b]		11.99	2 [b]	
K	9.33	1.04		8.40	0.91		6.29	0.80		8.70	0.98		5.27	0.60	
Fe	28.18	3.18		27.28	2.94		21.06	2.67		26.75	3.02		17.44	1.97	
Al+Fe	30.52	3.44		27.37	2.95		21.19	2.69		28.34	3.2		18.37	2.08	

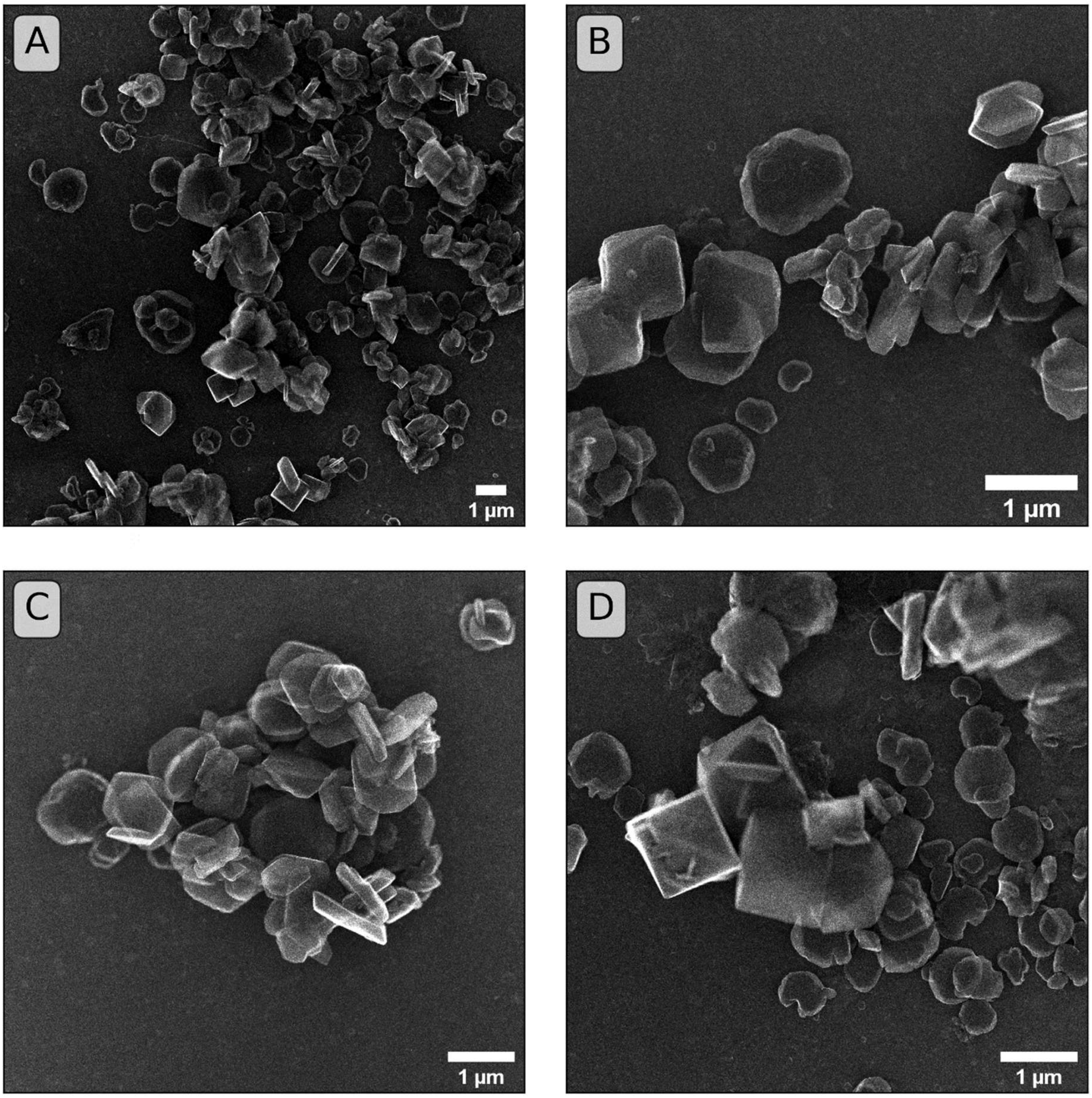

Figure S6: Secondary electron (SE) images of natural jarosite. Images of synthetic minerals are found in Figure 1 (main article).

3 Mineral colours

Table S2: Colours of jarosite samples investigated in this work, as determined by comparison of dried mineral samples with a physical Munsell colour chart. For quick comparison, sample colour squares for these colours are provided in the table from an online reference.[1, 2] To ensure accurate colour visualisation, the reader is advised to view this table in digital format on a calibrated display, or examine a certified physical reference.

Sample	Colour square	Munsell colour number	RGB value corresponding to Munsell colour	Notes
HT 0%		2.5Y 7/8	[209, 168, 72]	
HT 2%		2.5Y 7/8	[209, 168, 72]	
HT 5%		2.5Y 7/8	[209, 168, 72]	
HT 10%		2.5Y 7/8	[209, 168, 72]	
HT 15%		2.5Y 7/8	[209, 168, 72]	
HT 20%		2.5Y 7/8	[209, 168, 72]	
HT 25%		2.5Y 7/8	[209, 168, 72]	
HT 30%		2.5Y 8/8	[237, 195, 97]	
HT 40%		2.5Y 8/8	[237, 195, 97]	
16d RT 0%		5Y 8/6	[220,200,118]	True colour had higher chroma than any available physical reference
16d RT 5%		5Y 8/6	[220,200,118]	
16d RT 10%		5Y 8/6	[220,200,118]	
16d RT 20%		5Y 8/6	[220,200,118]	
16d RT 30%		5Y 8/6	[220,200,118]	
16d RT 40%		5Y 8/6	[220,200,118]	
16d RT 50%		2.5Y 8/6	[229,196,123]	
16d RT 60%		2.5Y 8/6	[229,196,123]	
16d RT 80%		7.5YR 7/6	[215,164,112]	
Natural		7.5Y 8/3	[208, 201, 157]	Illustration colour square in this table is of 7.5Y 8/4

4 Elemental analysis

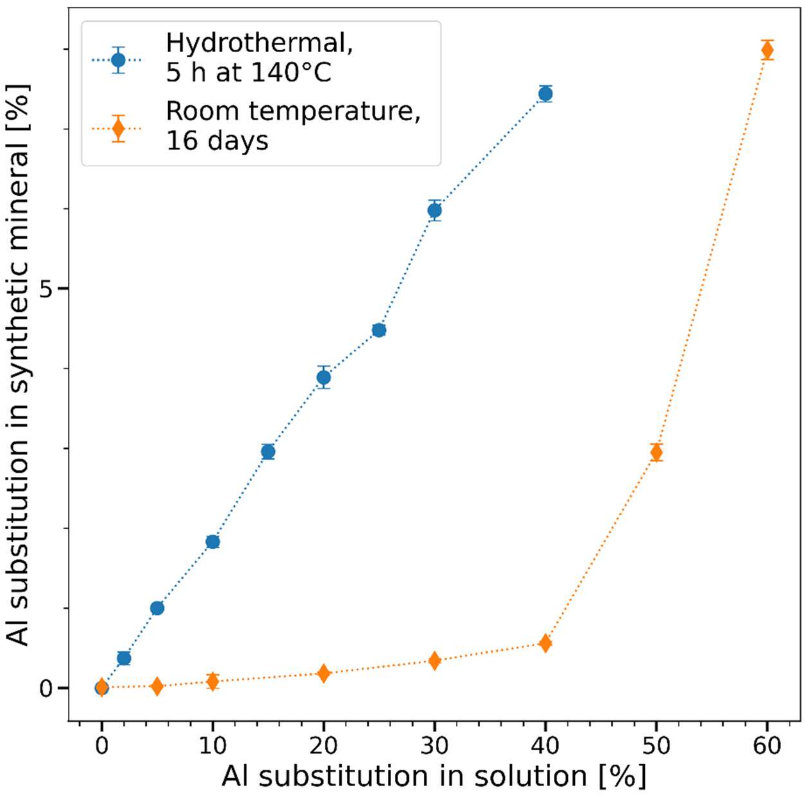

Figure S7: Substitution of Al in synthetic jarosite-alunite minerals as related to the Al content of the starting solution.

Table S3: Elemental analysis of jarosite samples by ICP-OES and AAS, reported as the ratios of each element to the theoretical 2 mol/mol S content of jarosite. [a] Jarosite synthesised by the hydrothermal (HT) and room temperature (RT) methods are identified by the ratio of Al to Fe in the synthesis solution. [b] The recovery is the sum of measured Al, Fe, K and S concentrations, normalised to the initial mass of dissolved mineral, plus the theoretical content of O (44%). [c] The Al-for-Fe substitution is calculated by $100 \times (\frac{Al}{Al+Fe})$.

Label[a]	Recovery[b]	Al	Fe	Al+Fe	K	S	Al-for-Fe [%] [c]	Al:S	Fe:S	Al+Fe :S	K:S
HT 0%	0.94	0.00	2.62 *±0.223*	2.62	0.80 *±0.094*	2 *±0.245*	0.00	0.00	1.31	1.31	0.40
HT 2%	0.95	0.01 *±0.002*	2.60 *±0.115*	2.61	0.82 *±0.074*	2 *±0.128*	0.42 *±0.078*	0.01	1.30	1.31	0.41
HT 5%	0.95	0.03 *±0.002*	2.65 *±0.130*	2.68	0.83 *±0.082*	2 *±0.139*	1.14 *±0.031*	0.02	1.33	1.34	0.42
HT 10%	0.95	0.06 *±0.003*	2.54 *±0.116*	2.60	0.85 *±0.075*	2 *±0.128*	2.15 *±0.066*	0.03	1.27	1.30	0.43
HT 15%	0.94	0.09 *±0.003*	2.59 *±0.071*	2.68	0.86 *±0.060*	2 *±0.085*	3.42 *±0.088*	0.05	1.30	1.34	0.43
HT 20%	0.95	0.12 *±0.007*	2.47 *±0.105*	2.59	0.85 *±0.073*	2 *±0.119*	4.69 *±0.135*	0.06	1.24	1.30	0.43
HT 25%	0.95	0.14 *±0.004*	2.44 *±0.057*	2.58	0.87 *±0.064*	2 *±0.064*	5.45 *±0.062*	0.07	1.22	1.29	0.44
HT 30%	0.95	0.19 *±0.005*	2.44 *±0.063*	2.63	0.86 *±0.057*	2 *±0.076*	7.26 *±0.128*	0.10	1.22	1.32	0.43
HT 40%	0.93	0.24 *±0.005*	2.29 *±0.048*	2.54	0.87 *±0.058*	2 *±0.056*	9.51 *±0.095*	0.12	1.15	1.27	0.44
RT 0%	0.91	0.00 *±0.000*	2.26 *±0.024*	2.26	0.70 *±0.023*	2 *±0.026*	0.01 *±0.019*	0.00	1.13	1.13	0.35
RT 5%	0.91	0.00 *±0.000*	2.27 *±0.036*	2.27	0.62 *±0.017*	2 *±0.039*	0.02 *±0.010*	0.00	1.14	1.14	0.31
RT 10%	0.89	0.00 *±0.000*	2.23 *±0.249*	2.23	0.57 *±0.054*	2 *±0.264*	0.08 *±0.061*	0.00	1.12	1.12	0.29
RT 20%	0.92	0.00 *±0.000*	2.21 *±0.029*	2.21	0.59 *±0.018*	2 *±0.032*	0.18 *±0.019*	0.00	1.11	1.11	0.30
RT 30%	0.91	0.01 *±0.000*	2.19 *±0.051*	2.20	0.59 *±0.017*	2 *±0.054*	0.34 *±0.024*	0.01	1.10	1.10	0.30
RT 40%	0.92	0.01 *±0.000*	2.14 *±0.082*	2.16	0.64 *±0.045*	2 *±0.085*	0.56 *±0.021*	0.01	1.07	1.08	0.32
RT 50%	0.92	0.07 *±0.004*	2.32 *±0.059*	2.39	0.67 *±0.025*	2 *±0.068*	2.95 *±0.138*	0.04	1.16	1.20	0.34
RT 60%	0.92	0.28 *±0.007*	3.22 *±0.023*	3.50	0.56 *±0.009*	2 *±0.039*	7.99 *±0.170*	0.14	1.61	1.75	0.28
Natural	0.86	0.26 *±0.011*	3.07 *±0.123*	3.33	0.86 *±0.052*	2 ±0.142	7.94 ±0.058	0.13	1.54	1.67	0.43

5 X-ray diffraction

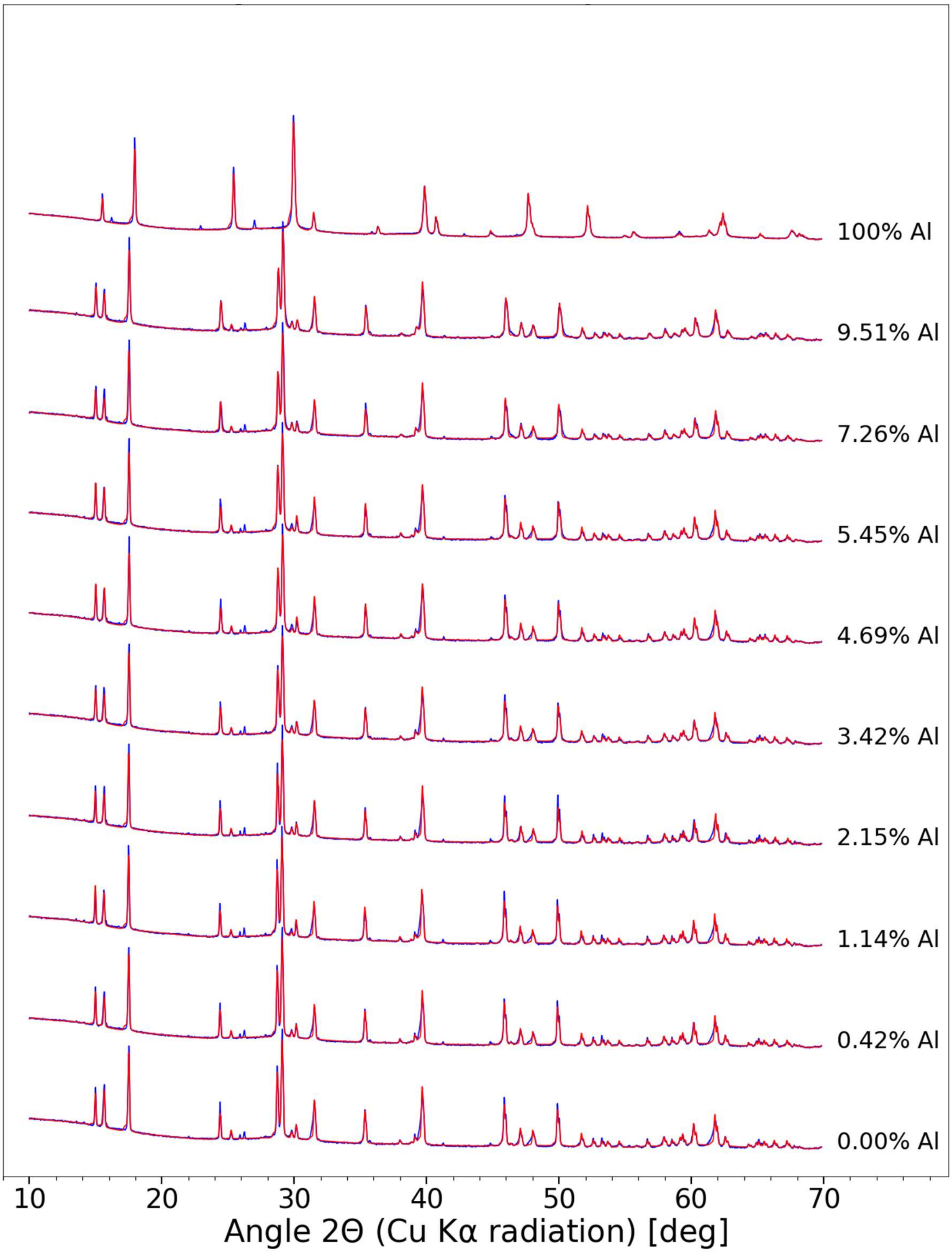

Figure S8: Fitted XRD patterns of hydrothermally synthesised jarosite minerals. Blue lines are plots of measured data and red is the best fit using a single jarosite or alunite model structure, as described in the main text.

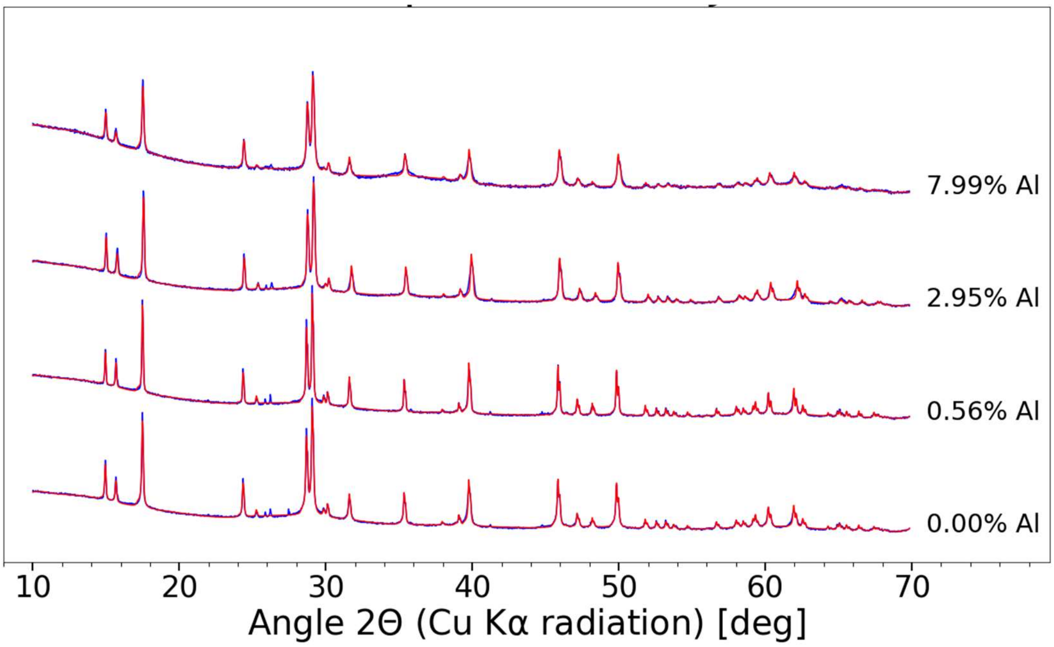

Figure S9: Fitted XRD patterns of jarosite minerals synthesised at room temperature. Blue lines are plots of measured data and red is the best fit using a single jarosite or alunite model structure, as described in the text.

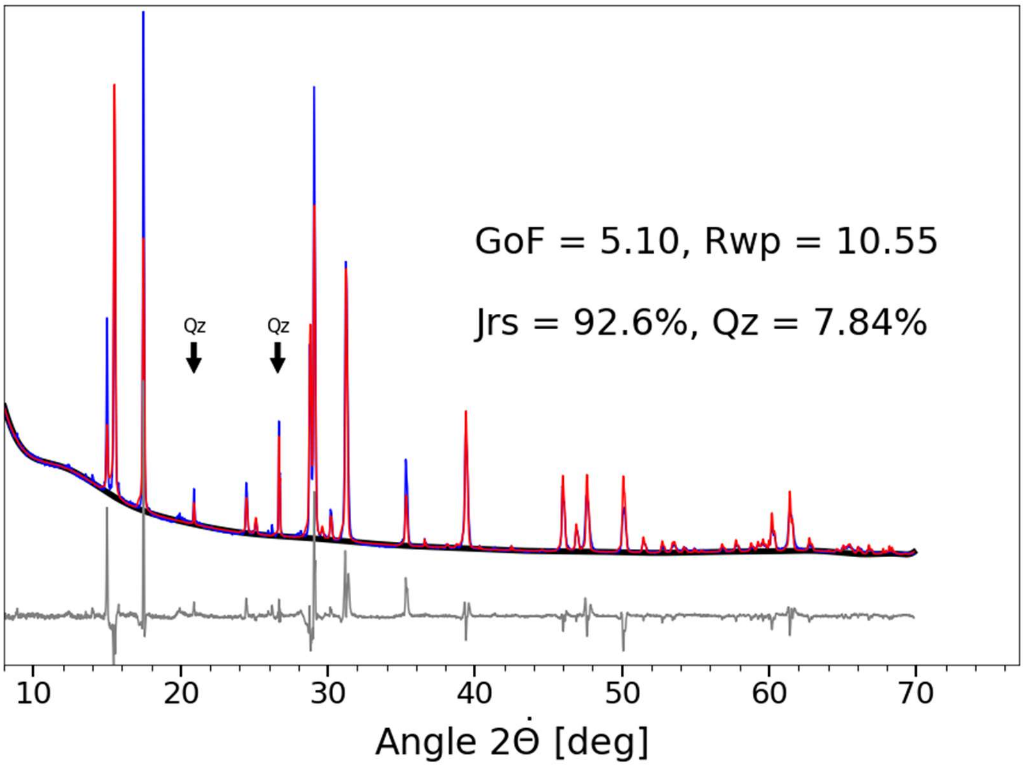

Figure S10: Rietveld fit of the XRD pattern for natural jarosite. Blue lines are plots of measured data and red is the best fit using model jarosite and quartz (Qz) structures. Additionally, the background is shown in black and the residual in grey. The goodness of fit (GoF) and Rwp are indicated on the plot with the estimated phase proportions.

Table S4: Parameters of the Rietveld fitting of XRD patterns of HT-Jrs, RT-Jrs and natural jarosite. The table presents unit cell sizes and crystallite sizes for all jarosite samples, as well as goodness-of-fit and Rwp as indicators of the fitting quality. All data and fitted curves are also plotted in Figures S8, S9 and S10. Each sample is identified by the ratio of Al to Fe in the synthesis solution (nominal substitution), with measured substitution also indicated (Figure 3). [a] Corrections in the right-hand columns were carried out as described in the main text, assuming H_3O-for-K substitution to the level of measured K deficiency and full Fe occupancy.

**Sample**	**Measured Al-for-Fe substitution (ICP-OES)**	**Goodness of fit**	**Rwp**	**c**	**a**	**Corrected a param. for B-site substitution [a]**	**Lvol-IB (nm)**
HT 0%	0.00	5.07	11.22	17.05560	7.314171	7.30224	95.311
HT 2%	0.42	4.85	10.79	17.05373	7.313757	7.30320	101.749
HT 5%	1.14	5.35	11.87	17.05765	7.311022	7.30090	103.490
HT 10%	2.15	6.51	14.59	17.05093	7.309184	7.30006	101.735
HT 15%	3.42	4.60	10.24	17.06003	7.306362	7.29770	82.849
HT 20%	4.69	5.01	11.10	17.05851	7.304422	7.29549	89.897
HT 25%	5.45	5.14	11.27	17.05417	7.302808	7.29508	92.397
HT 30%	7.26	7.35	16.13	17.05714	7.298482	7.29020	87.755
HT 40%	9.51	4.58	10.07	17.05523	7.293546	7.28593	75.837
HT Alunite	100	4.86	9.51	17.07046	7.019885	7.00489	82.598
16d RT 0%	0.00	2.78	6.04	16.99416	7.316686	7.29869	101.146
16d RT 40%	0.56	2.56	6.02	16.99367	7.317111	7.29551	136.889
16d RT 50%	2.95	3.28	7.07	16.93400	7.305977	7.28618	85.647
16d RT 60%	7.99	1.98	4.57	16.99500	7.301504	7.27510	64.918
Natural	-	5.10	10.55	17.18926	7.284350	-	82.725

6 Raman spectroscopy

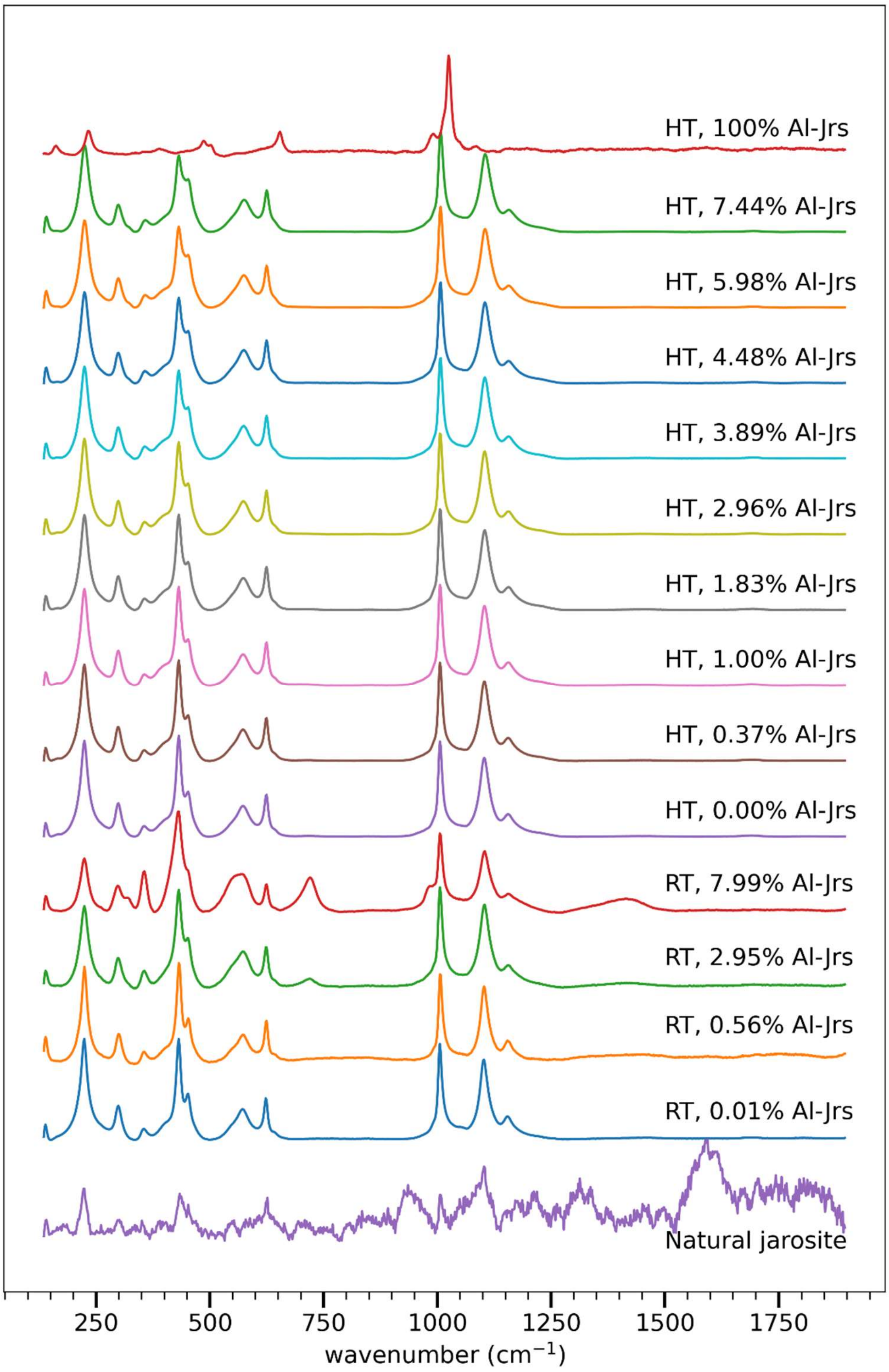

Figure S11: Raman spectra of all HT-Jrs, RT-Jrs and natural jarosite samples. Spectra were measured using the 1800 l/mm grating for display of spectra along a maximum possible domain. All spectra are normalised to their maximum range (peak intensity) for comparison. The natural jarosite spectrum is the average of two spectra measured using the method described in the main text. Spectra that were measured with a 3000 l/mm grating, used for the determination of peak locations (Table S5), are not plotted in this document.

Table S5: Peak positions of Raman spectra of room-temperature jarosite (RT-Jrs) and hydrothermal jarosite (HT-Jrs), measured using 3000 l mm^{-1} grating. Not all fitted peaks are reported, but the largest peaks are listed. Peak assignment is based on Maubec et al [3]. [a] The relationship between the Al substitution and measured peak locations are analysed by linear regression with gradient, m, and coefficient of determination, R^2. [b] Regressions exclude samples with 0% and 0.37% Al substitution, as they are outliers. Alunite was excluded from all regression calculations. [c] These peaks were present in jarosite and attributable to previously reported peaks but did not match observed peaks in alunite (see explanation below). [d] May be associated with another O/OH-B vibration as identified in jarosite samples (see explanation below).

Synth. series	Nominal % Al	Measured % Al	χ2	Peak positions [cm^{-1}]							
Peak attribution [3]				O-B$_{(1)}$ / frame	O-B$_{(2)}$	OH-B$_{(3)}$	$\nu_2(SO_4)$	O/OH-B$_{(4)}$	O/OH-B$_{(5)}$	$\nu_4(SO_4)$	$\nu_1(SO_4)$
HT-Jrs	0	0.00	12.2229	223.37	298.28	355.20	430.99	452.43	571.27	624.01	1006.1
	2	0.37	15.9190	223.35	298.16	355.04	430.71	452.58	571.29	623.92	1006.0
	5	1.00	14.6274	223.50	298.24	355.33	430.64	452.91	571.43	624.06	1006.2
	10	1.83	19.5884	223.69	298.39	355.72	430.62	453.19	571.78	624.25	1006.3
	15	2.96	19.0741	223.76	298.46	356.01	430.50	453.40	572.01	624.36	1006.4
	20	3.89	18.4411	224.01	298.57	356.54	430.46	453.67	572.47	624.57	1006.7
	25	4.48	19.4866	224.14	298.61	356.84	430.48	453.72	572.77	624.71	1006.8
	30	5.98	19.1973	224.31	298.73	357.41	430.39	454.02	573.04	624.93	1007.1
	40	7.44	24.3015	224.52	298.86	357.93	430.35	454.08	573.82	625.26	1007.4
	100	100	9.91106	232.96	- [c]	- [c]	487.07	502.17	567.94 [d]	654.13	1025.0
HT-Jrs regression	m [cm^{-1} $\%_{Al}^{-1}$] [a]			0.156	0.085	0.410	-0.072	0.230	0.294	0.196	0.188[b]
	R^2 [a]			0.98	0.95	0.99	0.90	0.98	0.95	0.90	0.99 [b]
RT-Jrs	0	0.01	12.0428	223.11	298.53	355.02	431.19	452.22	571.82	623.50	1006.0
	40	0.56	4.47411	222.86	298.31	354.95	431.56	452.49	572.38	623.81	1005.9
	50	2.95	6.51006	223.92	298.73	355.69	431.15	452.89	573.17	624.08	1006.3
	60	7.99	3.81633	223.92	296.80	355.23	430.66	452.77	578.06	624.37	1006.2
Natural jarosite from Thailand			0.815087	221.98	299.33	355.97	432.19	449.01	578.184	624.49	1007.0

Table abbreviations: **χ2:** Reduced chi-squared of the fit used to derive the peak positions. **O-B$_{(1)}$ / frame:** O-Fe stretching and/or framework deformation. **O-B$_{(2)}$:** O-Fe stretching. **O-B$_{(3)}$:** O-Fe stretching. **O/OH-B$_{(4)}$:** O-Fe and OH deformation. **O/OH-B$_{(5)}$:** O-Fe and OH deformation.

The measured peaks for Al-substituted jarosite were well matched to previously reported peak locations for jarosite.[3] The major peaks in the alunite sample could be attributed to previously reported peak locations for alunite.[3] Exceptions occurred for the 'O-B$_{(1)}$ / framework vibration' and 'O-B$_{(2)}$' peaks (Table S5), which were previously measured in alunite samples at 345 cm^{-1} and 381 cm^{-1}, respectively.[3] In our alunite sample, three small peaks occurred in this range, at 327.00 cm^{-1}, 360.97 cm^{-1} and 389.78 cm^{-1}, and none could be attributed to the previously identified peaks. Another exception occurred for the 'O/OH-B$_{(5)}$' peak. In jarosite, this peak corresponds to a peak that was previously observed near 573 cm^{-1}.[3] The alunite peak that we observed at 567.94 cm^{-1} has been placed in the same column in Table S5, but may be associated with a different vibration mode. Another O/OH-B vibration, which we did not observe in our jarosite samples, has previously been identified in jarosite near 551 cm^{-1}.[3] The peak measured in alunite at 567.94 cm^{-1} may be related to the 551 cm^{-1} vibration in jarosite.

7 Mössbauer spectroscopy

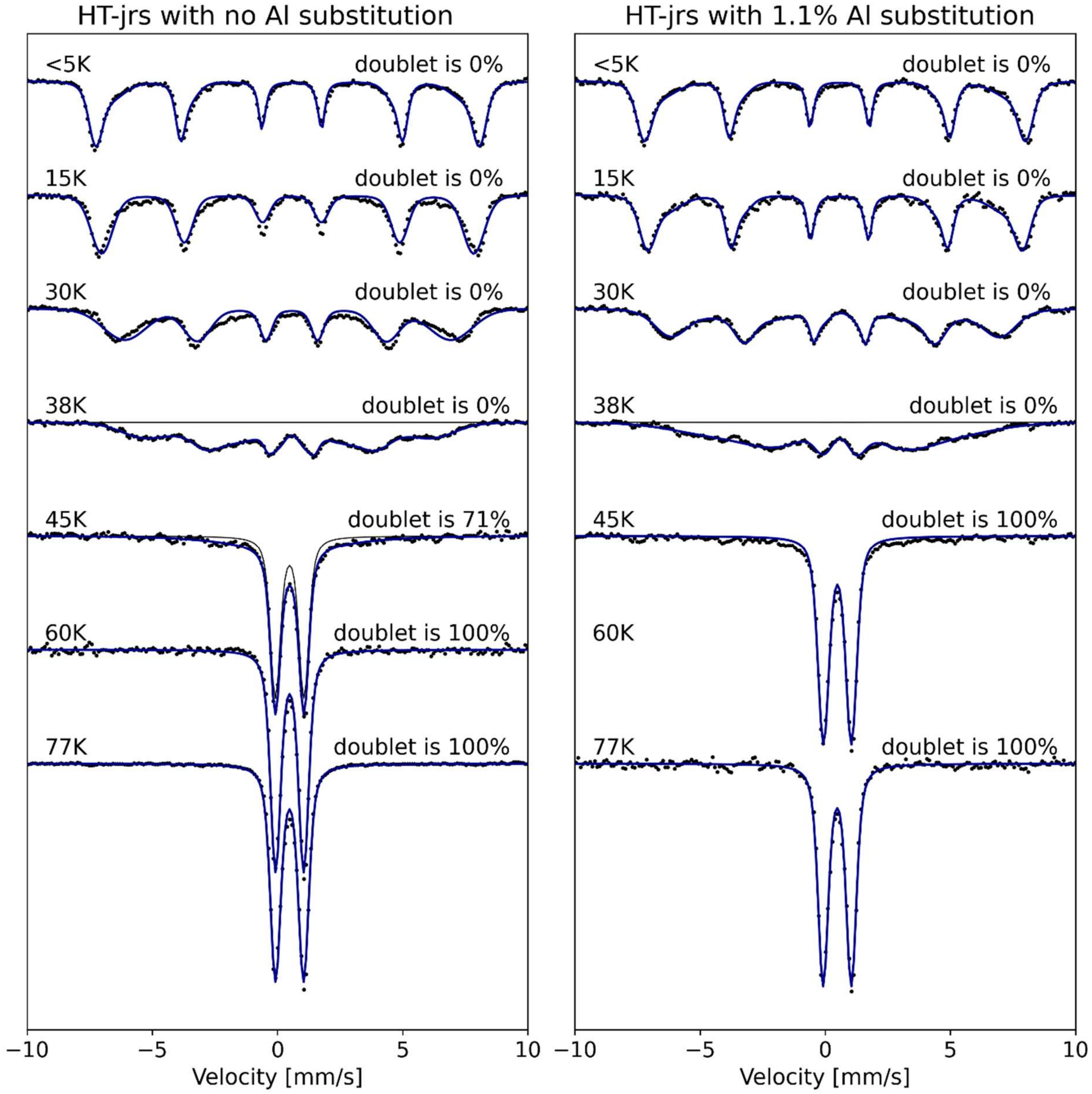

Figure S12: Mössbauer fits for synthetic hydrothermal jarosite with two levels of Al substitution (low substitution) at a series of temperatures between 4.2 K and 77 K. The doublet components of the fits of jarosite undergoing magnetic ordering are superimposed in grey on total spectra. All plots are normalised by integrated area of the respective fitted curve. Further plots of Mössbauer spectral fits are available in Figure S13, S14 and S15, line plots of the relative doublet/sextet areas are presented in Figure 6 and spectral data is available in Table 1 (main text) and Table S6.

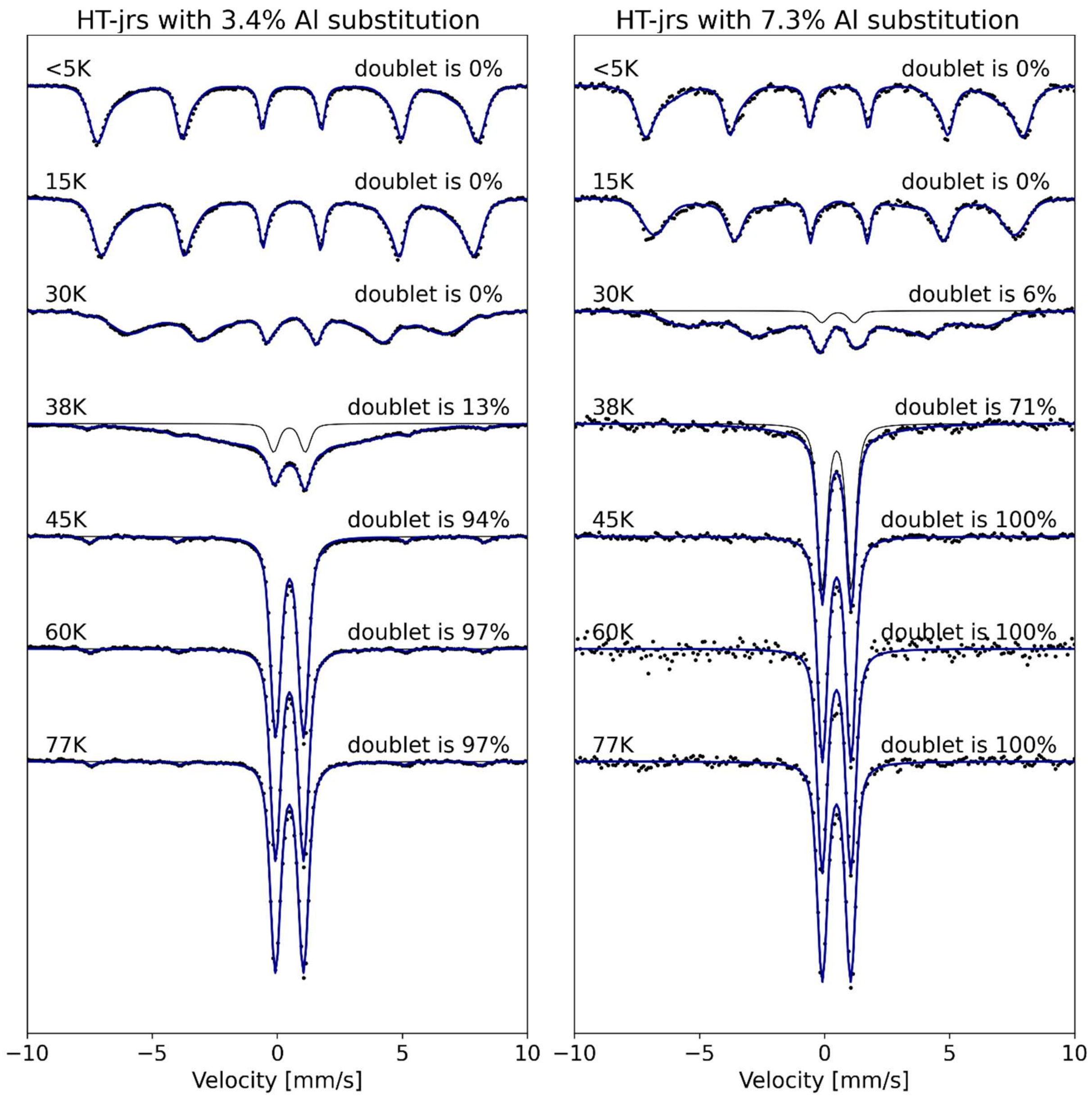

Figure S13: Mössbauer fits for synthetic hydrothermal jarosite with two levels of Al substitution (high Al substitution) at a series of temperatures between 4.2 K and 77 K. The doublet components of the fits of jarosite undergoing magnetic ordering are superimposed in grey on total spectra. All plots are normalised by integrated area of the respective fitted curve. Further plots of Mössbauer spectral fits are available in Figure S12 S14, and S15, line plots of the relative doublet/sextet areas are presented in Figure 6 and spectral data is available in Table 1 (main text) and Table S6.

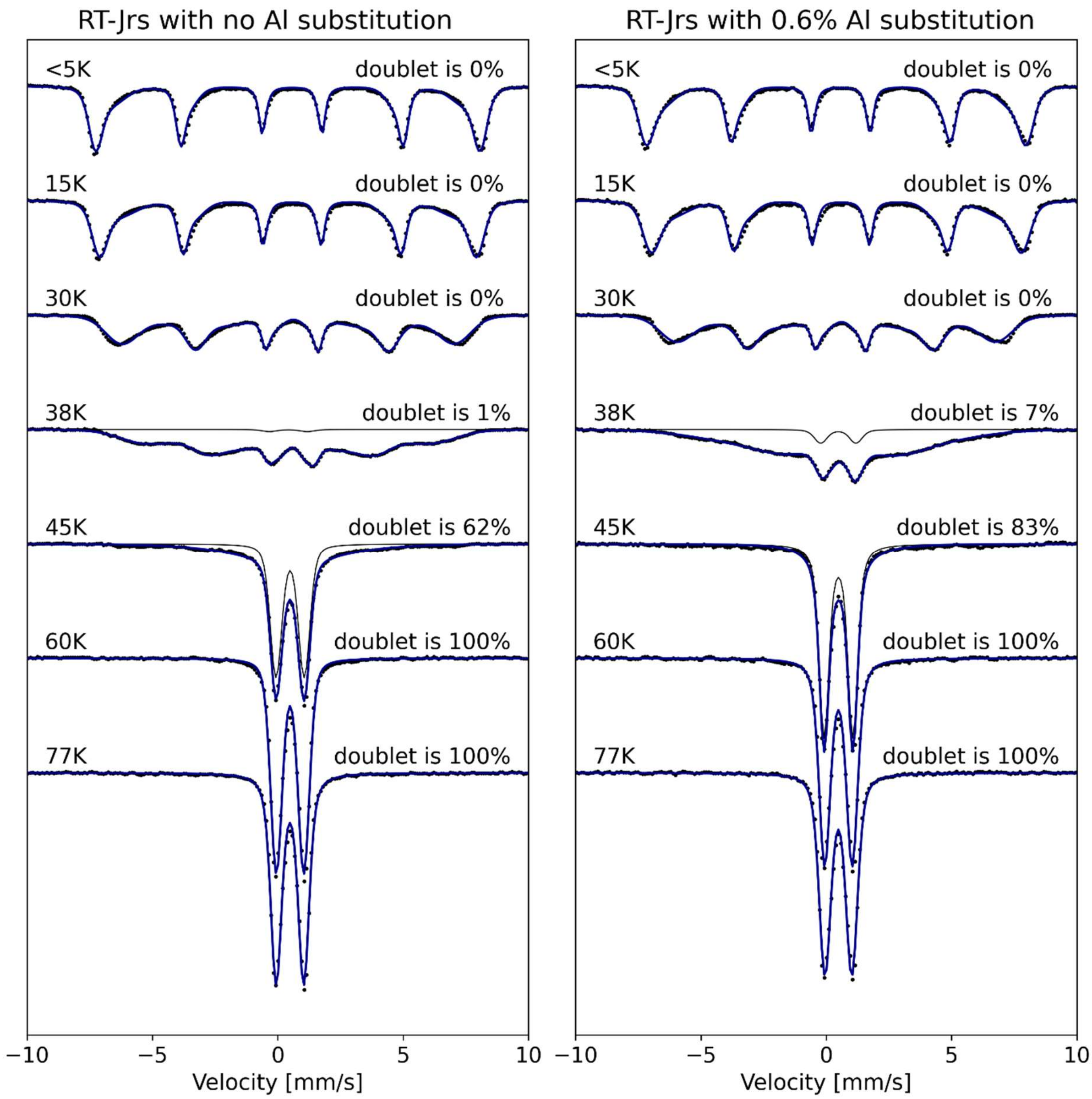

Figure S14: Mössbauer fits for synthetic room temperature jarosite with two levels of Al-for-Fe substitution at a series of temperatures between 4.2 K and 77 K. The doublet components of the fits of jarosite undergoing magnetic ordering are superimposed in grey on total spectra. All plots are normalised by integrated area of the respective fitted curve. Further plots of Mössbauer spectral fits are available in Figure S12, S13 and S15, line plots of the relative doublet/sextet areas are presented in Figure 6 and spectral data is available in Table 1 (main text) and Table S6.

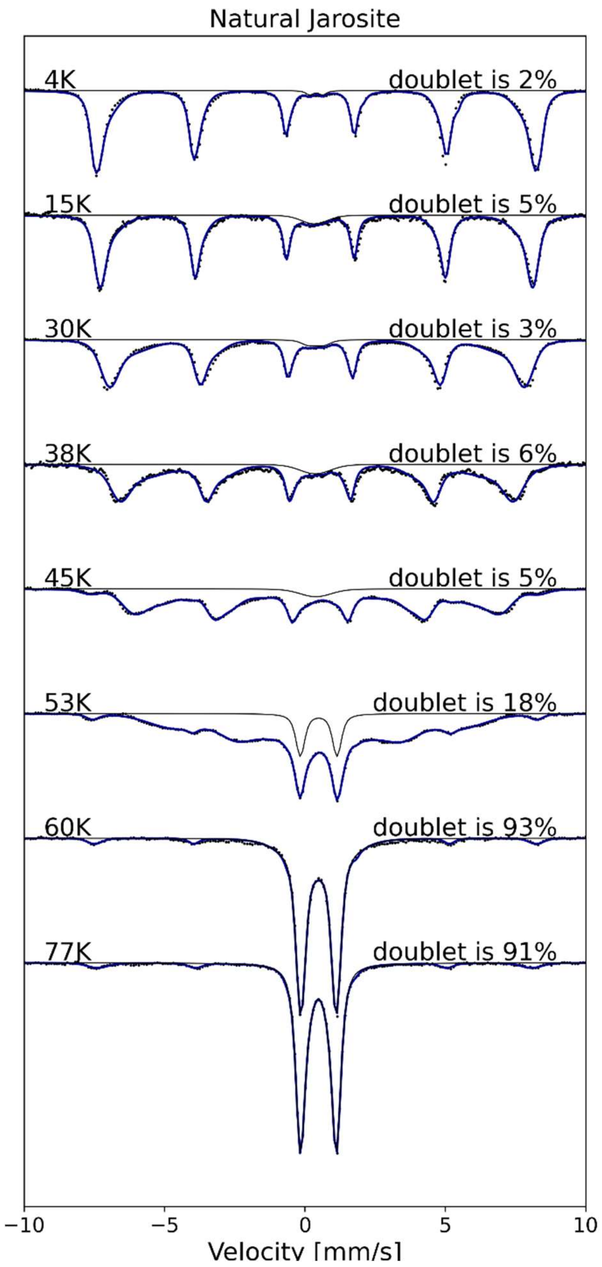

Figure S15: Mössbauer fits for natural jarosite at a series of temperatures between 4.2 K and 77 K. The doublet components of the fits of jarosite undergoing magnetic ordering are superimposed in grey on total spectra. All plots are normalised by integrated area of the respective fitted curve.

Table S6: Fitting parameters of all Mössbauer spectra plotted in Figures S12, S13, S14 and S15. Parameters for ≤5 K and 77 K spectra are also presented in Table 1. The parameters reported for each fitted phase are: Proportional area of the measured curve (Rel. spec. area), the centre-shift (CS), quadrupole splitting (QS) or quadrupole shift (ε), distribution of QS or ε (one standard deviation; σ_{QS} or σ_{ε}), hyperfine field (H) and distribution of the hyperfine field (one standard deviation; σ_H). When sextets were fitted with two hyperfine field components, the relative proportion of the first component is listed in the column marked 'P', and the parameters of the components from the first and second components are denotated as 'H_1' with 'σ_{H_1}' and 'H_2' with 'σ_{H_2}', respectively. Red- χ^2 is the reduced chi squared statistic to estimate the goodness of fit. Uncertainty of the last quoted significant figure is notated in parentheses or replaced with an asterisk (*) when the parameter was not floated during the fit. Uncertainties were calculated using the covariance matrix. Parameters that were not applicable to a particular component are fixed as 0*. Other than jarosite (Jrs), mineral phases identified in natural jarosite samples were goethite/ferrihydrite ('Gt/Fh'),[4] a paramagnetic Fe(II) soil phase consistent with Fe in phyllosilicate clays or organic complexes ('soil Fe(III)'),[5, 6] and collapsed features associated with minerals undergoing magnetic ordering ('partially ordered'). [a] Two techniques were applied to fit natural jarosite spectra at 77 K and 60 K, as described in the main text, and denoted here as fit v1 and v2.

Sample	Temp [K]	Interp-retation	Rel. spec. area [%]	CS [mm/s]	QS or ε [mm/s]	σ_{QS} or σ_{ε}	P [%]	H_1 [T]	σ_{H_1}	H_2 [T]	σ_{H_2}	Red-χ^2
**HT-Jrs 0% Al**	4	Jrs	100	0.4891(35)	-0.0810(35)	0*	34.7(76)	47.559(44)	1.304(99)	43.80(88)	3.27(44)	4.05
**HT-Jrs 0% Al**	15	Jrs	100	0.4942(67)	-0.0837(67)	0*	100*	46.025(59)	2.386(66)	0*	0*	4.13
**HT-Jrs 0% Al**	30	Jrs	100	0.495(11)	-0.078(10)	0*	100*	40.421(99)	4.915(96)	0*	0*	7.22
**HT-Jrs 0% Al**	38	Jrs	49.2(51)	0.516(58)	-0.056(54)	0*	100*	19.1(15)	7.82(95)	0*	0*	0.60
**HT-Jrs 0% Al**	38	Jrs	50.8(51)	0.493(33)	-0.073(31)	0*	100*	35.39(65)	5.50(47)	0*	0*	0.60
**HT-Jrs 0% Al**	45	Jrs	70.9(26)	0.4871(41)	1.1462(87)	0.254(15)	-	-	-	-	-	0.88
**HT-Jrs 0% Al**	45	Jrs	29.1(26)	0.5*	0*	0*	100*	0(170)	16(53)	0*	0*	0.88
**HT-Jrs 0% Al**	60	Jrs	100	0.4876(35)	1.1247(67)	0.2765(88)	-	-	-	-	-	1.22
**HT-Jrs 0% Al**	77	Jrs	100	0.48396(88)	1.1230(17)	0.2890(22)	-	-	-	-	-	11.17
**HT-Jrs 1.1% Al**	4	Jrs	100	0.4847(59)	-0.0918(59)	0*	35.4(95)	47.262(76)	1.49(15)	43.3(12)	3.86(57)	1.92
**HT-Jrs 1.1% Al**	15	Jrs	100	0.4814(100)	-0.0878(99)	0*	37(22)	46.50(14)	1.61(31)	42.0(27)	3.7(14)	1.10
**HT-Jrs 1.1% Al**	30	Jrs	54.9(37)	0.490(14)	-0.086(14)	0*	100*	40.85(14)	3.45(24)	0*	0*	1.05
**HT-Jrs 1.1% Al**	30	Jrs	45.1(37)	0.526(76)	-0.119(71)	0*	100*	31.2(15)	10.52(84)	0*	0*	1.05
**HT-Jrs 1.1% Al**	38	Jrs	0.02(69)	0.49*	1.1*	0.2*	-	-	-	-	-	1.32
**HT-Jrs 1.1% Al**	38	Jrs	99.98(69)	0.470(42)	-0.131(39)	0*	100*	26.14(35)	11.72(33)	0*	0*	1.32
**HT-Jrs 1.1% Al**	45	Jrs	100	0.4890(23)	1.1150(44)	0.3158(55)	-	-	-	-	-	3.51
**HT-Jrs 1.1% Al**	77	Jrs	100	0.4821(36)	1.1253(69)	0.2770(91)	-	-	-	-	-	1.20

Sample	Temp [K]	Interp-retation	Rel. spec. area [%]	CS [mm/s]	QS or ε [mm/s]	σ_{QS} or σ_ε	P [%]	H_1 [T]	σ_{H_1}	H_2 [T]	σ_{H_2}	Red-χ^2
**HT-Jrs 3.4% Al**	5	Jrs	100	0.4931(24)	-0.0921(24)	0*	41.4(33)	47.198(34)	1.512(61)	43.60(37)	3.96(15)	7.05
**HT-Jrs 3.4% Al**	15	Jrs	80.2(23)	0.4993(34)	-0.0827(34)	0*	56(10)	42.86(83)	3.38(44)	46.307(58)	1.56(13)	4.09
**HT-Jrs 3.4% Al**	15	Jrs	19.8(23)	0.56(10)	-0.196(97)	0*	100*	33.3(19)	11.8(11)	0*	0*	4.09
**HT-Jrs 3.4% Al**	30	Jrs	47.0(35)	0.4889(97)	-0.0862(94)	0*	100*	39.287(99)	4.06(22)	0*	0*	2.25
**HT-Jrs 3.4% Al**	30	Jrs	51.0(35)	0.536(35)	-0.099(33)	0*	100*	28.5(14)	11.13(62)	0*	0*	2.25
**HT-Jrs 3.4% Al**	30	Jrs	1.99(76)	0.493(48)	-0.093(48)	0*	100*	50.28(39)	0.70(65)	0*	0*	2.25
**HT-Jrs 3.4% Al**	38	Jrs	13.25(51)	0.4869(62)	1.264(12)	0.281(20)	-	-	-	-	-	2.70
**HT-Jrs 3.4% Al**	38	Jrs	81.51(72)	0.519(37)	-0.132(38)	0*	100*	18.16(26)	14.82(43)	0*	0*	2.70
**HT-Jrs 3.4% Al**	38	Jrs	5.24(61)	0.451(33)	-0.146(33)	0*	100*	49.58(29)	1.87(37)	0*	0*	2.70
**HT-Jrs 3.4% Al**	45	Jrs	93.78(56)	0.4947(12)	1.1211(22)	0.2908(29)	-	-	-	-	-	6.29
**HT-Jrs 3.4% Al**	45	Jrs	6.22(56)	0.472(26)	-0.085(26)	0*	100*	49.00(22)	1.06(28)	0*	0*	6.29
**HT-Jrs 3.4% Al**	60	Jrs	97.34(51)	0.4907(11)	1.1227(21)	0.2820(27)	-	-	-	-	-	7.18
**HT-Jrs 3.4% Al**	60	Jrs	2.66(51)	0.486(41)	-0.103(41)	0*	100*	48.69(31)	0.46(54)	0*	0*	7.18
**HT-Jrs 3.4% Al**	77	Jrs	100	0.4874(11)	1.1219(22)	0.2819(28)	-	-	-	-	-	6.74
**HT-Jrs 7.3% Al**	4	Jrs	100	0.4846(93)	-0.0927(92)	0*	54.7(79)	46.85(13)	1.45(22)	43.88(70)	4.41(35)	1.22
**HT-Jrs 7.3% Al**	15	Jrs	100	0.487(14)	-0.090(14)	0*	36.5(48)	44.66(13)	2.79(18)	32.6(26)	12.4(17)	1.11
**HT-Jrs 7.3% Al**	30	Jrs	5.9(20)	0.546(90)	1.300(93)	0.33(18)	-	-	-	-	-	0.65
**HT-Jrs 7.3% Al**	30	Jrs	29.3(87)	0.518(45)	-0.111(45)	0*	100*	37.46(46)	4.17(96)	0*	0*	0.65
**HT-Jrs 7.3% Al**	30	Jrs	64.8(85)	0.487(98)	-0.059(88)	0*	100*	23.8(28)	11.4(14)	0*	0*	0.65
**HT-Jrs 7.3% Al**	38	Jrs	70.6(29)	0.4905(49)	1.156(10)	0.235(17)	-	-	-	-	-	0.74
**HT-Jrs 7.3% Al**	38	Jrs	29.4(29)	0.48(35)	-0.23(41)	0*	100*	0(2100)	20(250)	0*	0*	0.74
**HT-Jrs 7.3% Al**	45	Jrs	100	0.4880(25)	1.1421(49)	0.2605(66)	-	-	-	-	-	1.73
**HT-Jrs 7.3% Al**	60	Jrs	100	0.4830(85)	1.146(17)	0.264(22)	-	-	-	-	-	0.62
**HT-Jrs 7.3% Al**	77	Jrs	100	0.4809(41)	1.1358(79)	0.276(10)	-	-	-	-	-	1.05
**RT-Jrs 0% Al**	5	Jrs	100	0.4900(15)	-0.0832(15)	0*	37.3(28)	47.583(18)	1.267(40)	43.81(31)	3.37(16)	15.01
**RT-Jrs 0% Al**	15	Jrs	100	0.4932(16)	-0.0790(16)	0*	38.1(23)	46.641(19)	1.522(39)	41.76(32)	4.15(17)	18.77
**RT-Jrs 0% Al**	30	Jrs	38.7(11)	0.489(20)	-0.145(19)	0*	100*	29.21(53)	10.16(30)	0*	0*	15.68
**RT-Jrs 0% Al**	30	Jrs	61.3(11)	0.4949(31)	-0.0768(32)	0*	100*	41.251(30)	3.594(59)	0*	0*	15.68

Sample	Temp [K]	Interp-retation	Rel. spec. area [%]	CS [mm/s]	QS or ε [mm/s]	σ_{QS} or σ_{ε}	P [%]	H_1 [T]	σ_{H_1}	H_2 [T]	σ_{H_2}	Red-χ^2
**RT-Jrs 0% Al**	38	Jrs	1.90(69)	0.446(98)	1.67(18)	0.53(23)	-	-	-	-	-	1.33
**RT-Jrs 0% Al**	38	Jrs	66.2(50)	0.470(20)	-0.122(21)	0*	100*	21.4(13)	10.43(75)	0*	0*	1.33
**RT-Jrs 0% Al**	38	Jrs	31.9(50)	0.499(17)	-0.073(15)	0*	100*	36.38(31)	5.36(40)	0*	0*	1.33
**RT-Jrs 0% Al**	45	Jrs	62.00(62)	0.4935(11)	1.1258(24)	0.3695(45)	-	-	-	-	-	7.27
**RT-Jrs 0% Al**	45	Jrs	38.00(62)	0.505(48)	-0.037(55)	0*	100*	0(300000)	18(58)	0*	0*	7.27
**RT-Jrs 0% Al**	60	Jrs	100	0.49122(83)	1.0955(16)	0.3723(18)	-	-	-	-	-	19.05
**RT-Jrs 0% Al**	77	Jrs	100	0.48696(80)	1.1005(15)	0.3090(19)	-	-	-	-	-	15.87
**RT-Jrs 0.6% Al**	5	Jrs	100	0.4908(20)	-0.0882(20)	0*	43.0(40)	47.050(32)	1.444(63)	43.75(35)	3.40(15)	9.61
**RT-Jrs 0.6% Al**	15	Jrs	100	0.4982(32)	-0.0842(31)	0*	38.7(54)	45.862(41)	1.737(89)	40.96(71)	4.15(35)	6.78
**RT-Jrs 0.6% Al**	30	Jrs	56.8(20)	0.4902(62)	-0.0882(63)	0*	100*	39.631(61)	3.63(12)	0*	0*	4.86
**RT-Jrs 0.6% Al**	30	Jrs	43.2(20)	0.487(35)	-0.132(33)	0*	100*	26.51(94)	10.51(53)	0*	0*	4.86
**RT-Jrs 0.6% Al**	38	Jrs	7.31(70)	0.483(20)	1.404(31)	0.375(52)	-	-	-	-	-	1.24
**RT-Jrs 0.6% Al**	38	Jrs	92.69(70)	0.443(29)	-0.133(26)	0*	100*	20.97(23)	12.48(26)	0*	0*	1.24
**RT-Jrs 0.6% Al**	45	Jrs	82.83(93)	0.4905(14)	1.1460(28)	0.2476(47)	-	-	-	-	-	5.34
**RT-Jrs 0.6% Al**	45	Jrs	17.17(93)	0.657(75)	0.214(98)	0*	100*	0(75000)	9(64)	0*	0*	5.34
**RT-Jrs 0.6% Al**	60	Jrs	100	0.4878(13)	1.0960(25)	0.3203(31)	-	-	-	-	-	8.40
**RT-Jrs 0.6% Al**	77	Jrs	100	0.4860(13)	1.0714(24)	0.3364(29)	-	-	-	-	-	8.35
**Thai Nat-Jrs**	4	Soil Fe(III)	1.39(15)	0.399(23)	0.470(42)	0*	-	-	-	-	-	22.43
**Thai Nat-Jrs**	4	Jrs	88.48(54)	0.4808(13)	-0.0643(13)	0*	35.3(44)	48.386(20)	0.818(49)	46.14(31)	2.17(14)	22.43
**Thai Nat-Jrs**	4	Gt/Fh	10.14(53)	0.6332(59)	-0.1958(57)	0*	100*	49.456(54)	0.19(15)	0*	0*	22.43
**Thai Nat-Jrs**	15	Soil Fe(III)	4.59(52)	0.342(66)	0(250000)	1(14)	-	-	-	-	-	1.47
**Thai Nat-Jrs**	15	Jrs + Gt/Fh	95.41(52)	0.4843(28)	-0.0690(28)	0*	33.7(54)	47.789(38)	0.850(70)	45.71(45)	2.60(21)	1.47
**Thai Nat-Jrs**	30	Soil Fe(III)	3.36(20)	0.387(33)	0.536(52)	0.419(96)	-	-	-	-	-	15.63
**Thai Nat-Jrs**	30	Jrs + Gt/Fh	96.64(20)	0.4945(20)	-0.0574(20)	0*	38.8(29)	45.869(27)	1.569(51)	41.76(37)	4.08(16)	15.63
**Thai Nat-Jrs**	38	Soil Fe(III)	5.08(42)	0.391(49)	0.568(70)	0.4*	-	-	-	-	-	1.73
**Thai Nat-Jrs**	38	Jrs + Gt/Fh	94.92(42)	0.4889(65)	-0.0587(63)	0*	55.2(40)	43.438(84)	1.73(13)	39.40(53)	5.63(24)	1.73
**Thai Nat-Jrs**	45	Soil Fe(III)	4.3(10)	0.476(38)	0.4(26)	0.9(15)	-	-	-	-	-	4.80
**Thai Nat-Jrs**	45	Jrs	88.90(94)	0.4926(45)	-0.0569(43)	0*	45(10)	41.06(11)	1.80(19)	36.09(65)	3.60(54)	4.80

Sample	Temp [K]	Interp-retation	Rel. spec. area [%]	CS [mm/s]	QS or ε [mm/s]	σ_{QS} or σ_{ε}	P [%]	H_1 [T]	σ_{H_1}	H_2 [T]	σ_{H_2}	Red-χ^2
**Thai Nat-Jrs**	45	Gt/Fh	6.84(11)	0.442(21)	-0.112(21)	0*	100*	49.36(20)	1.89(19)	0*	0*	4.80
**Thai Nat-Jrs**	53	Jrs	12.66(85)	0.4934(42)	1.3051(82)	0.174(12)	-	-	-	-	-	1.36
**Thai Nat-Jrs**	53	Gt/Fh	4.36(45)	0.472(20)	-0.123(20)	0*	100*	49.02(17)	1.04(22)	0*	0*	1.36
**Thai Nat-Jrs**	53	Jrs	51.6(32)	0.508(28)	-0.069(26)	0*	100*	15.0(11)	9.68(81)	0*	0*	1.36
**Thai Nat-Jrs**	53	Jrs	31.4(35)	0.479(17)	-0.045(15)	0*	100*	31.93(40)	5.33(30)	0*	0*	1.36
**Thai Nat-Jrs**	60 (v1) [a]	Jrs	42.6(25)	0.4861(20)	1.2842(51)	0.194(11)	-	-	-	-	-	0.68
**Thai Nat-Jrs**	60 (v1) [a]	Jrs	31.0(26)	0.4926(74)	1.146(21)	0.495(27)	-	-	-	-	-	0.68
**Thai Nat-Jrs**	60 (v1) [a]	Gt/Fh	6.23(63)	0.478(23)	-0.102(23)	0*	100*	48.86(20)	1.25(23)	0*	0*	0.68
**Thai Nat-Jrs**	60 (v1) [a]	Partially ordered	20.2(14)	0.5*	0*	0*	-	-	-	-	-	0.68
**Thai Nat-Jrs**	60 (v2) [a]	Jrs	72.5(17)	0.4864(12)	1.2691(26)	0.2038(41)	-	-	-	-	-	0.87
**Thai Nat-Jrs**	60 (v2) [a]	Soil Fe(III)	4.85(99)	0.45*	0.46*	0.27*	-	-	-	-	-	0.87
**Thai Nat-Jrs**	60 (v2) [a]	Jrs	6.70(63)	0.480(23)	-0.104(23)	0*	100*	48.84(19)	1.05(26)	0*	0*	0.87
**Thai Nat-Jrs**	60 (v2) [a]	Partially ordered	16.0(17)	0.70(11)	0*	0*	100*	11.0(35)	12.3(41)	0*	0*	0.87
**Thai Nat-Jrs**	77 (v1) [a]	Jrs	67.8(29)	0.48405(100)	1.2869(45)	0.1658(91)	-	-	-	-	-	0.89
**Thai Nat-Jrs**	77 (v1) [a]	Jrs	23.7(31)	0.484282*	0.972(58)	0.437(23)	-	-	-	-	-	0.89
**Thai Nat-Jrs**	77 (v1) [a]	Gt/Fh	8.54(75)	0.460(28)	-0.115(28)	0*	100*	47.91(24)	1.92(28)	0*	0*	0.89
**Thai Nat-Jrs**	77 (v2) [a]	Jrs	84.96(61)	0.48319(90)	1.2676(20)	0.1992(30)	-	-	-	-	-	1.30
**Thai Nat-Jrs**	77 (v2) [a]	Soil Fe(III)	7.36(34)	0.45*	0.46*	0.27*	-	-	-	-	-	1.30
**Thai Nat-Jrs**	77 (v2) [a]	Gt/Fh	7.68(57)	0.455(28)	-0.114(28)	0*	100*	47.97(24)	1.67(28)	0*	0*	1.30

8 References

1. Grackle Studio Yellow - Grackle Studio. accessed from https://www.grackle.studio/yellow/ on 9 August 2022.
2. Grackle Studio Yellow Red - Grackle Studio. Accessed from https://www.grackle.studio/yellow-red/ on 9 August 2022.
3. Maubec, N.; Lahfid, A.; Lerouge, C.; Wille, G.; Michel, K., Characterization of alunite supergroup minerals by Raman spectroscopy. *Spectrochim. Acta Part A* **2012,** *96*, 925–939.
4. Notini, L.; ThomasArrigo, L. K.; Kaegi, R.; Kretzschmar, R., Coexisting goethite promotes Fe(II)-catalyzed transformation of ferrihydrite to goethite. *Environ. Sci. Technol.* **2022,** *56*, (17), 12723–12733.
5. Murad, E., Clays and clay minerals: What can Mössbauer spectroscopy do to help understand them? *Hyperfine Interact.* **1998,** *117*, 39–70.
6. Kodama, H.; Schnitzer, M.; Murad, E., An investigation of iron(III)-fulvic acid complexes by Mössbauer spectroscopy and chemical methods. *Soil Sci. Soc. Am. J.* **1988,** *52*, (4), 903–1204.
